# Relationship between Fish Consumption and Undernutrition among Young Indian Children

**DOI:** 10.1016/j.cdnut.2025.107610

**Published:** 2025-11-29

**Authors:** Rajesh Kumar Rai, Sabri Bromage, Baban Bayan, Baishnaba Charan Ratha, Rockli Kim, Sourabh Kumar Dubey, Wanjiku N Gichohi-Wainaina, Edward H Allison, Cristiano M Rossignoli, Arun Panemangalore Padiyar, S.V. Subramanian, Christopher D Golden

**Affiliations:** 1 Birbhum Population Project, Society for Health and Demographic Surveillance, Suri, West Bengal, India; 2 Division of Global Health Management & Policy, School of Public Health, San Diego State University, San Diego, CA, United States; 3 Human Nutrition Unit, Institute of Nutrition, Mahidol University, Phutthamonthon, Nakhon Pathom, Thailand; 4 Department of Global Health and Population, Harvard T H Chan School of Public Health, Harvard University, Boston, MA, United States; 5 Community Nutrition Unit, Institute of Nutrition, Mahidol University, Phutthamonthon, Nakhon Pathom, Thailand; 6 Department of Nutrition, Harvard T H Chan School of Public Health, Harvard University, Boston, MA, United States; 7 WorldFish, New Delhi, India; 8 Division of Health Policy & Management, College of Health Science, Korea University, Seoul, Korea; 9 WorldFish, Jalan Batu Maung, Penang, Malaysia; 10 Harvard Center for Population and Development Studies, Harvard University, Cambridge, MA, United States; 11 Department of Social and Behavioral Sciences, Harvard T H Chan School of Public Health, Harvard University, Boston, MA, United States; 12 Department of Environmental Health, Harvard T H Chan School of Public Health, Harvard University, Boston, MA, United States

**Keywords:** aquatic food, fish, dietary diversity, child anthropometry, anemia, India

## Abstract

**Background:**

Although aquatic foods, particularly fish, hold promise as a dietary intervention to address child undernutrition, evidence supporting their role in Indian context remains limited and mixed, thus inconclusive.

**Objectives:**

We assessed the association between fish consumption and undernutrition among children aged 6–23 mo using data from India's nationally representative, cross-sectional 2019–2021 National Family Health Survey (NFHS-5). This survey covered 707 districts across 36 states/union territories.

**Methods:**

Undernutrition indicators included stunting (sample: 59,560), wasting (sample: 59,145), underweight (sample: 61,450), any anthropometric failure (sample: 60,440), and anemia (sample: 58,850). Stunting, wasting, and underweight were defined as <–2 standard deviations from the median of z-scores for height-for-age, weight-for-height, and weight-for-age of the reference population, respectively. Children measured as being either stunted, wasted, or underweight were defined as having any anthropometric failure. Children were classified as having any anemia, based on hemoglobin levels of <10.5 g/dL. Children's 24-h dietary recall provided information on fish consumption. We used modified Poisson regression models with robust error variance to attain the study objective.

**Results:**

Nationally, a strikingly low prevalence of fish consumption among young children was recorded, with only 4.5% of them reporting intake in the preceding 24 h. Children in 111 of the 707 districts surveyed in NFHS-5 reported no fish consumption. Regression analysis revealed no association (*P* > 0.1) between fish consumption and anthropometric indicators (stunting, wasting, underweight, and any anthropometric failure) among children aged 6–23 mo. However, a protective association of fish consumption with anemia was observed (relative risk: 0.947; 95% confidence interval: 0.921, 0.973; *P* < 0.001).

**Conclusion:**

Encouraging fish consumption through public initiatives like the National Nutrition Mission could be a valuable strategy for mitigating the burden of anemia among young children. Further research utilizing detailed food consumption data, including fish intake, is warranted to investigate its impact on child health.

## Introduction

Child undernutrition in India remains a pressing concern [1] with a high prevalence of stunting (36%), wasting (19%), and underweight (32%) among children under 5, whereas a staggering 67% of children aged 6–59 mo are affected by some degree of anemia (as estimated in 2019–2021) [2]. Stunting, a consequence of long-term nutritional deprivation or recurrent infections, can hinder cognitive development, leading to poor school performance and reduced intellectual capacity, ultimately impacting economic productivity; wasting, resulting from insufficient food intake or a high incidence of infectious diseases, impairs the functioning of the immune system, increasing a child's vulnerability to illness; whereas even mild underweight increases risk of mortality among children [3,4]. Anemia, with its diverse causes [5] poses a serious threat to child development, impacting cognitive development and, in severe cases, leading to mortality [4]. Although the Indian government has launched initiatives such as the National Nutrition Strategy (in 2017) and the National Nutrition Mission or Prime Minister's Overarching Scheme for Holistic Nutrition *Abhiyaan* (in 2018) to combat child undernutrition, these efforts have not yet yielded a discernible reduction in undernutrition rates, underscoring the need for intensified action [1].

Of potential interventions, promoting fish consumption, an excellent source of animal protein, essential omega-3 fatty acids, and bioavailable micronutrients [6,7], represents a highly promising strategy to reduce the burden of child undernutrition [[8], [9], [10], [11]]. However, this benefit must be weighed against the health implications of exposure to dioxins, methylmercury, and other contaminants present in fish [12]. With rising fish production (from 6.574 metric tons in 2005–2006 to 14.164 metric tons in 2019–2020) and increasing annual per capita consumption (from 4.9 to 8.89 kg during the same period) [13], India has the potential to leverage its abundant aquatic resources, particularly fish, to combat child undernutrition [13,14]. Furthermore, the *Pradhan Mantri Matsya Sampada Yojana*, which was launched by the Department of Fisheries, Government of India, in financial year 2020–2021, ensures the healthy, economically viable, and socially inclusive development of India's fisheries sector [15], thereby potentially contributing to the reduction of child undernutrition. To ensure equitable access to this valuable nutritional resource, program and policymakers must address the complex web of social, economic, environmental, religious, and gender barriers that prevent many children in India from consuming fish.

Although fish is a rich source of essential nutrients, the current evidence base on its impact on child undernutrition remains limited and mixed, and thus inconclusive [16]. A study [17] of 130,432 children aged 6–23 mo from 49 countries concluded that meat/fish consumption was associated with a reduced risk of stunting, but the study did not disaggregate the findings by meat and fish consumption. Similarly, a cross-sectional study in Zambia [18] observed a protective association between fish consumption and reduced stunting. Although some impact evaluations have found positive effects on stunting/linear growth [19,20], others have found no significant difference between fish-based interventions and control groups [21]. In Malawi, a study found a reduced likelihood of stunting, wasting, and underweight among children from fish-farming households compared with those from nonfish-farming households [22]. Similar to the findings on anthropometric outcomes, the evidence on the role of fish or fish-based products in improving iron status or reducing anemia is inconclusive [16]. Two trials conducted in Cambodia [19] and Kenya [23] suggested an overall deterioration in iron status over time, regardless of the intervention. This may be attributed to chronic inflammation that can impair iron absorption, or other untreated infections [5].

It is important to acknowledge the limitations that may contribute to these observed variations in the relationship between fish consumption and child undernutrition. These limitations include variations in study design, the type of fish intervention, potential measurement errors in assessing nutritional status, and the influence of unobservable confounding factors. Overall, the current evidence on the relationship between fish consumption and child undernutrition, particularly in the Indian context, is limited and premature. To fill this knowledge gap, this study utilized a nationally representative dataset to investigate the association between fish consumption and undernutrition (stunting, wasting, underweight, any anthropometric failure, and anemia) among young children aged 6–23 mo in India. Focusing the analysis on the infants and young children (aged 6–23 mo)–comprising both breastfed and nonbreastfed children–holds crucial implications for reducing risk of growth faltering and nutrient deficiencies and, consequently, the associated morbidity and mortality [24].

## Methods

### Dataset and sample selection

We used data from 3 waves of the cross-sectional nationally representative National Family Health Survey (NFHS): NFHS-3 (2005–2006) [25], NFHS-4 (2015–2016) [26], and NFHS-5 (2019–2021) [2]. As data on exclusive fish consumption among children were unavailable in NFHS-1 (1992–1993) [27] and NFHS-2 (1998–1999) [28], both waves were excluded from this study. Although data from all 3 rounds of the NFHS (2005–2021) were used to analyze trends in fish intake among children by state and union territory, only the NFHS-5 dataset (covering 707 districts across 36 states and union territories) was used to examine the relationship between fish consumption and undernutrition among children aged 6–23 mo. Because of their consistent sampling design, estimates across NFHS rounds are comparable [29]. The Ministry of Health and Family Welfare, Government of India, entrusted the International Institute for Population Sciences (IIPS) in Mumbai with the responsibility of conducting the NFHSs. Findings from the NFHS play a crucial role in shaping public health and population policies in India, providing valuable data for researchers, donors, and policymakers. NFHSs employed a stratified 2-stage sampling design, with primary sampling units selected in rural areas and census enumeration blocks in urban areas. This rigorous survey methodology secured a household response rate of over 97%. Detailed information regarding the sampling design and process, sample weighting computation, estimation of SEs, and strategies to enhance data quality can be found in the respective NFHS reports [2,25,26].

All NFHSs utilize 4 questionnaires: household, man, woman, and biomarker. Information on fish consumption among children was gathered from mothers through the woman's questionnaire, whereas data on child anthropometry and hemoglobin levels were collected using the biomarker questionnaire. NFHS-5 covered 232,920 children aged 0–59 mo. Of these, 224,218 were reported to be alive, and food intake data (including fish intake) was recorded for the youngest child (sample: 127,648). This study analyzed nutritional deficiencies in young children (aged 6–23 mo) using the following sample sizes: 59,560 for stunting, 59,145 for wasting, 61,450 for underweight, 60,440 for any anthropometric failure, and 58,850 for anemia. The flowchart illustrating the steps of sample derivation is provided in Supplemental Figure 1.

### Indicators of child undernutrition

Measurements of child anthropometry and hemoglobin levels followed the guidelines established by the WHO. Following WHO growth reference standards [30], 3 nutritional z-scores were computed for children aged 6–23 mo: height-for-age (HAZ), weight-for-height (WHZ), and weight-for-age (WAZ). Stunting, wasting, and underweight were defined as <–2 SDs from the median HAZ, WHZ, and WAZ of the reference population, respectively. Severe stunting, severe wasting, and severe underweight were defined as <–3 SDs from the median HAZ, WHZ, and WAZ of the reference population, respectively. Children measured as being either stunted, wasted, or underweight were defined as having any anthropometric failure [31]. In NFHS-5 [2], weight was measured using a Seca 874 digital scale, height was measured using a Seca 213 stadiometer, and length (for children <2 y old or <85 cm) was measured using a Seca 417 infantometer.

Hemoglobin levels (recorded in g/dL) were measured in children aged 6–23 mo, adjusted for altitude of residence, following the revised WHO guidelines for defining anemia [32]. Children aged 6–23 mo were classified as having any anemia, mild, and moderate/severe anemia based on hemoglobin levels of <10.5 g/dL, 9.5–10.4 g/dL, and ≤9.4 g/dL, respectively. To mitigate the potential impact of measurement error on the analysis, any hemoglobin values exceeding 20 g/dL or falling below 4 g/dL were excluded [32]. NFHS-5 utilized on-site hemoglobin testing with a portable HemoCue Hb 201+ analyzer, assessing capillary blood obtained via finger prick [2]. Readers can find further details on the protocol for measuring child anthropometry and hemoglobin levels in the NFHS-5's published report [2].

### Measurement of fish consumption

**FIGURE 1:**
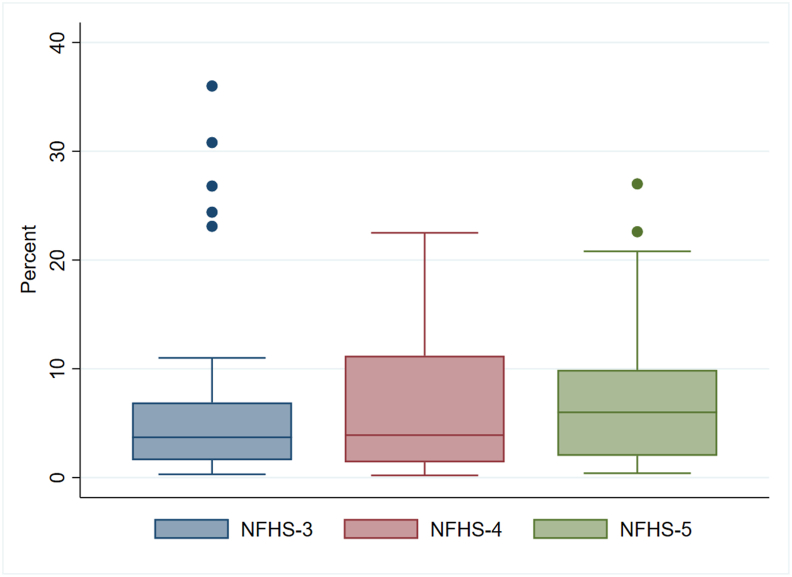
Summary distribution of the percentage of children who consumed fish by 29 states/union territories across National Family Health Survey (NFHS) waves—NFHS-3 (2005–2006), NFHS-4 (2015–2016), and NFHS-5 (2019–2021). See Supplemental Table 1 for further details on the restricted representation of 29 states/ UTs. The horizontal bar inside the box indicates the median (50th percentile). The lower and upper ends of the boxes represent the 25th and 75th percentiles, respectively, defining the IQR. The bottom “whisker” below the box is called the lower adjacent value and is equal to the 25th percentile minus 1.5 times the IQR. The upper “whisker” above the box is called the upper adjacent value and is equal to the 75th percentile plus 1.5 times the IQR. Circles indicate outliers.

In all NFHSs [2,25,26], mothers were asked on the day of the survey about “liquids or foods that (name of the child) had yesterday during the day or at night,” even if it was combined with other foods. This question applied to the youngest child living with their mother who was born in 2003 or later (in NFHS-3), 2014 or later (in NFHS-4), and 2018 or later (in NFHS-5). The list of food items included an option for “any fresh or dried fish or shellfish,” allowing for the recording of fish consumption. One of 3 possible responses were recorded—“yes,” “no,” or “don’t know.” Responses of “don’t know” were reported by a negligible proportion of respondents (<0.05%). These responses were treated as indicating that the child had not been given any type of fish. This approach, which has been used in previous studies [33,34], is not expected to affect the overall analysis. Although a single 24-h recall may not accurately represent a child’s typical diet or fish consumption, it offers valuable insight into population-level dietary trends, and this data collection method is less susceptible to recall errors [35]. Furthermore, these NFHS estimates on child feeding practices (based on 24-h recall) are widely used by the Indian government and international agencies like the WHO, UNICEF, and the World Bank to inform public health policies [36]. The prevalence of fish consumption among children remained virtually unchanged between 2005–2006 (NFHS-3) and 2019–2021 (NFHS-5), at 4.4% [95% confidence interval (CI): 4.2, 4.6] and 4.5% (95% CI: 4.4, 4.6), respectively, although a state-wise variation in coverage was observed (Figure 1 and Supplemental Table 1). Moreover, an analysis of the NFHS-5 data documented a broad district-wise range (Supplemental Table 2) and corresponding distribution (Supplemental Figure 2) regarding the prevalence of fish consumption among children residing in the 707 districts of the 36 states/union territories. It was also observed that although children in 111 districts consumed no fish, the highest reported rate of fish intake, nearly 38.6%, was documented in the East Garo Hills district of Meghalaya state. Supplemental Figure 3 represents kernel density distribution of HAZ, WHZ, WAZ, and hemoglobin level among children aged 6–23 mo, by their fish consumption status.

### Statistical approach

Descriptive statistics were used to estimate the proportional differences in stunting, wasting, underweight, any anthropometric failure, and anemia by child fish consumption status. To examine the relationship between fish consumption and child nutritional status, modified Poisson regression models with robust error variance [37] were used to estimate relative risks (RRs) and 95% CIs, adjusting for potential confounders. For the regression analyses, binary outcome variables were created for stunting, wasting, underweight, any anthropometric failure, and anemia, where each variable was coded as “1” if the condition was present and “0” otherwise.

The selection of potential confounders was guided by existing literature and data availability within the NFHS-5. Child anthropometry and anemia status in India could be influenced by various factors [1,3,5,31,[38], [39], [40], [41], [42], [43], [44], [45], [46], [47]] namely child’s individual-level characteristics [age, sex, birth order, food intake in last 24 h, experience of acute respiratory infection (ARI) and diarrhea preceding 2 wk of survey, and if the child was born with low birthweight (<2.5 kg), household characteristics (household size, religion, social group, wealth index, locality of residence: urban compared with rural, and state of residence], and mother’s characteristics [age, educational attainment, BMI (in kg/m^2^), tobacco use, alcohol use, hypertension status, and hyperglycemia status]. The study also explored the relationship between fish consumption and undernutrition by controlling covariates on utilization of maternal healthcare use including ≥4 antenatal care visits, ≥100 iron/folic acid supplementation, and delivery mode (cesarean section delivery compared with otherwise).

A 24-h dietary recall was conducted to determine the child's intake of various food groups. This included animal-source foods (excluding fish): poultry (chicken, duck, etc.), other meats, eggs, organ meats (heart, liver, etc.), and dairy products (yogurt, milk, etc.); carbohydrate-rich foods: grains—bread, noodles, and other grains, and tubers—potatoes, cassava, and other tubers; vegetables: pumpkin, carrots, yellow or orange squash; and dark leafy greens; fruits of any kind; and lentils or other pulses, and any solid/semisolid foods. An episode of ARI was defined as short, rapid breathing or difficult breathing related to the chest within 2 wk preceding the survey, and the same 2-wk period was used to assess episodes of diarrhea [2]. Social groups were categorized as Scheduled Caste, Scheduled Tribe, Other Backward Class, and Others, as defined by the Constitution of India [2]. State of residence was divided into 3 groups depending on the abundance of fish availability, namely states with access to marine fish (include island territories: Andaman & Nicobar Islands and Lakshadweep; coastal states: Gujarat, Maharashtra, Goa, Karnataka, Kerala, Tamil Nadu, Puducherry, Andhra Pradesh, Telangana, and Odisha), freshwater river fish (include North-Eastern States/Brahmaputra basin states, Gangetic basin states: Uttar Pradesh, Uttarakhand, Bihar, and West Bengal; tribal-dominated states such as Jharkhand, Chhattisgarh, and Madhya Pradesh), and remaining states/union territories with poor/other types of fish availability [13,48]. BMI for Asian Indians was categorized as follows: underweight (<18.5), normal weight (18.5–22.9), and overweight including obesity (≥23) [49]. To ensure methodological consistency with previous NFHS-5 studies, identical definitions were employed for both hypertension (elevated blood pressure) and hyperglycemia (elevated blood glucose) [48,50].

All analyses were performed using Stata version 18 [51]. We applied the sample weighting provided in the NFHS dataset when calculating descriptive statistics. Five regression models were developed to analyze the relationship between fish consumption and each outcome indicators namely stunting, wasting, underweight, any anthropometric failure, and anemia. Each model progressively incorporated more variables: Model I (Null Model): fish consumption only, Model II: fish consumption and child's individual characteristics, Model III: fish consumption, child's characteristics, and household characteristics, Model IV: all variables from Model III plus mother’s characteristics, and Model V: all variables from Model IV plus maternal healthcare utilization. By progressively incorporating key variables, this step-wise regression modeling approach illuminated the relationship between fish consumption and undernutrition in Indian children, effectively controlling for potential confounders. All reported *P* values are 2-tailed. Prior to executing modified Poisson regression, variance inflation factors (VIFs) [52] were calculated to assess multicollinearity among the predictor variables for each outcome variable (stunting, wasting, underweight, any anthropometric failure, and anemia). A VIF >5 indicated the presence of multicollinearity for that predictor variable. Missing values in select covariates were assigned a random value allowing us to include all cases in both descriptive and regression analyses, potentially reducing sample selection bias.

To provide further context to the study findings, we adopted a 2-pronged approach. First, we conducted additional analyses examining the association between fish intake and specific nutritional indicators among children aged 6–23 mo, including severe stunting, severe wasting, severe underweight, hemoglobin levels, mild anemia, and moderate/severe anemia. Second, we replicated the analysis investigating the association between fish consumption and child anthropometry, expanding the age group from the original 6–23 mo to 0–59 mo. Concurrently, the corresponding anemia analysis was repeated for children aged 6–59 mo. This methodological extension significantly broadened the study's scope, allowing us to evaluate the role of fish consumption in undernutrition indicators across a much wider population.

### Ethics approval

Prior to conducting the NFHSs, ethics approval was obtained by the implementing university, IIPS, Mumbai, India, from an independent ethics review committee constituted by the Ministry of Health and Family Welfare, Government of India. Thus, no separate ethics approval was required for this study.

## Results

### Descriptive statistics

**FIGURE 2:**
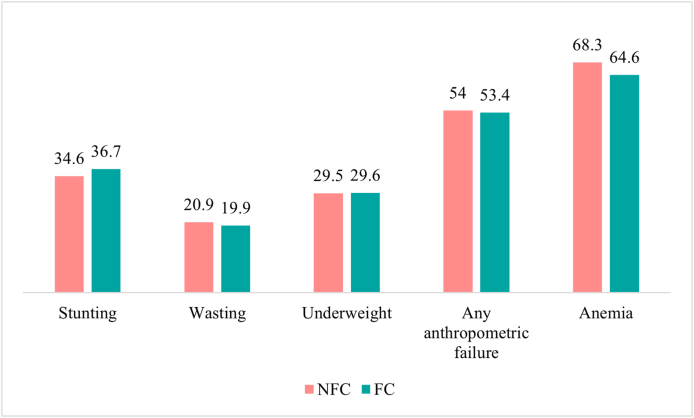
Prevalence of stunting, wasting, underweight, any anthropometric failure, and anemia in children aged 6–23 mo, categorized by fish consumption (FC) versus nonfish consumption (NFC).

Young children in the fish consumption (FC) group had a higher prevalence of stunting (36.7%; 95% CI: 35.1%, 38.3%) than those who did not [nonfish consumption (NFC)] (34.6%; 95% CI: 34.2%, 35%) (Figure 2 and Supplemental Table 3). However, there was 1 percentage point difference in the prevalence of wasting between the 2 groups (NFC: 20.9%, 95% CI: 20.5%, 21.2%; FC: 19.9%, 95% CI: 18.6%, 21.3%). The prevalence of underweight showed no apparent difference between the NFC and FC groups, at 29.5% (95% CI: 29.1%, 29.8%) and 29.6% (95% CI: 28.1%, 31.1%) (Figure 2 and Supplemental Table 4), respectively. Over 53% of children in both the FC (53.4%; 95% CI: 51.7%, 55.1%) and NFC (54%; 95% CI: 53.6%, 54.4%) groups were estimated to have ≥1 anthropometric failure. The prevalence of anemia was estimated to be higher among the NFC group (68.3%; 95% CI: 67.9%, 68.7%) than among the FC group (64.6%; 95% CI: 63%, 66.2%) (Figure 2 and Supplemental Table 5).

### Association between fish consumption and undernutrition

Multicollinearity was assessed using VIFs, and all VIFs were <5, indicating a low likelihood of substantial multicollinearity (data not shown separately). Although the final adjusted model (Model V) demonstrated no association between fish consumption and anthropometric indicators of undernutrition, the unadjusted model revealed a preliminary association with stunting (RR: 1.079; 95% CI: 1.034, 1.126; *P* = 0.001) and wasting (RR: 0.920; 95% CI: 0.859, 0.986; *P* = 0.018) (Table 1). This suggests that the observed association in the initial model may be attributable to confounding factors accounted for in the final model. Across all 5 models (model I through model V), a consistent protective association was observed between fish consumption and anemia (e.g., in model V—RR: 0.947; 95% CI: 0.921, 0.973; *P* < 0.001). Supplemental Table 6 further examined the association between fish consumption and a range of severe nutritional deficiencies, including severe stunting, severe wasting, severe underweight, mild, and moderate/severe anemia. The table also presents the association between fish consumption and hemoglobin level.

**TABLE 1:** Association between fish consumption and undernutrition among Indian children (aged 6–23 mo)

	Model I^1^	Model II^2^	Model III^3^	Model IV^4^	Model V^5^
	RR (95% CI) *P*	RR (95% CI) *P*	RR (95% CI) *P*	RR (95% CI) *P*	RR (95% CI) *P*
Stunting	1.079 (1.034, 1.126) 0.001	1.041 (0.994, 1.091) 0.086	1.011 (0.966, 1.059) 0.640	1.015 (0.969, 1.062) 0.539	1.014 (0.969, 1.062) 0.544
Wasting	0.920 (0.859, 0.986) 0.018	1.021 (0.948, 1.100) 0.584	1.006 (0.934, 1.085) 0.865	1.015 (0.942, 1.094) 0.692	1.016 (0.943, 1.095) 0.675
Underweight	0.967 (0.917, 1.019) 0.207	1.023 (0.967, 1.083) 0.422	1.002 (0.948, 1.060) 0.939	1.009 (0.955, 1.067) 0.741	1.010 (0.955, 1.067) 0.736
Any anthropometric failure	1.009 (0.979, 1.040) 0.565	1.025 (0.992, 1.059) 0.138	1.003 (0.971, 1.036) 0.850	1.008 (0.976, 1.041) 0.640	1.008 (0.976, 1.041) 0.620
Anemia	0.907 (0.883, 0.930) <0.001	0.920 (0.895, 0.946) <0.001	0.944 (0.919, 0.971) <0.001	0.947 (0.921, 0.973) <0.001	0.947 (0.921, 0.973) <0.001

Abbreviations: CI, confidence interval; RR, relative risk.

1 Null model.

2 Adjusted for individual characteristics.

3 Adjusted for individual and household characteristics.

4 Adjusted for individual, household, and maternal characteristics.

5 Adjusted for individual, household, maternal, and maternal healthcare use characteristics

Furthermore, the analysis was repeated using broader age groups to assess both child anthropometry (0–59 mo) and anemia (6–59 mo). Supplemental Figure 4 depicts the kernel density distribution for HAZ, WHZ, WAZ, and hemoglobin levels among children aged 0–59 mo, stratified by their fish consumption status. Conversely, Supplemental Figure 5 shows the prevalence of stunting, wasting, underweight, any anthropometric failure, and anemia, according to the children's reported fish intake. Regression analysis (Supplemental Table 7) revealed that, similar to children aged 6–23 mo, children in the 6–59 mo age group were less likely to be diagnosed with anemia if they reported consuming fish (RR: 0.962; 95% CI: 0.940, 0.984; *P* = 0.001). To execute the extended analysis, we redefined the definition of anemia for children aged 6–59 mo: a hemoglobin level of <10.5 g/dL for children aged 6–23 mo, and a hemoglobin level of <11.0 g/dL for children aged 24–59 mo were classified as anemic [32].

## Discussion

This study examined the relationship between fish intake and undernutrition in young Indian children aged 6–23 mo, using data from the 2019–2021 NFHS. We used modified Poisson regression models with robust error variance to examine the association between fish intake and undernutrition (stunting, wasting, underweight, any anthropometric failure, and anemia), adjusting for potential confounders. Contrary to expectations, the regression analysis revealed that fish consumption was not associated with anthropometric indicators, including stunting, wasting, underweight, and composite anthropometric failure. However, a protective association with anemia was observed.

The finding that fish consumption is not associated with anthropometry in children aged 6–23 mo (stunting, wasting, underweight, and composite anthropometric failure) should be interpreted with caution. The limitations of the fish consumption data, particularly the reliance on 24-h recall, may hinder the ability to identify a true association with child anthropometry. Future research should consider the complex interplay of factors contributing to anthropometric failure and employ more robust methods to assess fish consumption patterns. Additionally, although anthropometric indicators are important for monitoring nutritional status, they may not be the most sensitive or comprehensive measures for assessing the immediate impacts of nutrition-sensitive interventions (such as fish intake in last 24 h), which often aim to improve dietary practices and household economic well-being [16,53]. This hypothesis warrants further investigation in future studies, as it could have significant implications for interventions required to mitigate child undernutrition.

Results from the modified Poisson regression analysis demonstrated a protective association between fish consumption and reduced risk of anemia. Projected dietary shifts in India, with rising consumption of sugar, dairy, vegetables, and fruit, and consistent consumption of rice, wheat, and pulses, have significant implications for child nutrition [54], particularly regarding anemia where the bioavailability of consumed food is crucial. Flesh foods, such as fish, are rich in highly bioavailable heme iron, which can significantly enhance the absorption of nonheme iron from other foods consumed in the same meal, playing a crucial role in improving iron status among children [[55], [56], [57]]. Fresh fish also provides a small amount of vitamin C that is essential for iron absorption among children [58]. Our finding regarding the protective association of fish consumption with reduced anemia is consistent with a study conducted among Malawian children [59], which showed that fish consumption was associated with increased hemoglobin levels and a reduced prevalence of iron deficiency anemia.

Although these results offer valuable insights, it is essential to acknowledge the study's limitations. First, the definition of children's fish consumption in the NFHSs is broad, including all types of fresh and dried fish and shellfish (excluding marine mammals and algae), which precludes a separate analysis of different fish types and their potentially varying nutritional impacts. Second, the information collected on whether children consumed fish in the past 24 h is highly susceptible to unmeasured confounding variables, including the affordability, local availability, and accessibility of fish to the household. The absence of these crucial data points necessitates a prudent interpretation of the results derived from this study. Third, the NFHS does not include information on aspects of fish preparation such as cooking methods, ingredients used, or the use of sauces or spices when fish is given to children. Although protein and fat remain stable after cooking, some micronutrients are sensitive to fish processing and cooking methods [60]. Fourth, the reliance on self-reported data in the NFHS-5 (for information on households, men, women, and children) introduces the possibility of recall errors and social desirability bias, which may affect the accuracy of the findings. Fifth, the current approach of collecting fish consumption data based on 24-h recall is limited in its ability to capture the nuances of household dietary habits and preferences, particularly as the mother often determines the food choices for her children. Finally, the hemoglobin measurements used to calculate anemia were derived from capillary blood collected via finger pricks, rather than from a venous blood sample. This methodology is known to sometimes overestimate hemoglobin concentration [5]. In addition, the capillary blood samples were not adjusted for confounding factors such as the presence of inflammation or infectious diseases, rendering the hemoglobin measurements vulnerable to bias. This study, despite its limitations in capturing the full complexity of fish consumption patterns, contributes valuable findings on the association between fish consumption and child undernutrition in India.

Although analysis of NFHS-5 highlights the need for more comprehensive data on fish consumption, it also provides valuable evidence to support policies promoting fish consumption as a means of combating undernutrition, particularly anemia among children. However, further research is necessary to better understand the specific role of nutrients in various fish species on the health outcomes of Indian children. This could be achieved, perhaps, by developing nationwide cohorts and designing a dedicated cohort study to examine health outcomes based on children's fish consumption exposure. A more comprehensive approach, encompassing nationally representative details such as fish species, portion sizes, source, and preparation methods, would provide a more accurate understanding of nutrient intake and its association with health outcomes among Indian children. To move beyond improving exposure measurements (such as dietary intake estimates), future studies should prioritize more accurate evaluations of childhood undernutrition. By assessing micronutrient status and inflammation status alongside anemia and anthropometry, the research can afford a more granular understanding of the mechanisms (both dietary and nondietary) that influence anemia prevalence. In light of these findings, it is posited that a comprehensive framework should be established for the systematic integration of fish consumption into extant governmental public health programs, with the explicit goal of ameliorating the burden of anemia. This integration could be realized through strategic mechanisms such as: promoting piscine dietary inclusion among pediatric populations via established delivery channels like the Integrated Child Development Services program and the mid-day meal scheme, under the directive of the National Nutrition Mission, and provisioning fish or fish-derived nutritional supplements to expectant mothers to optimize perinatal health status and enhance the developmental trajectory of newborn infants [61,62]. The positive trend in India's fish consumption underscores the need for policies that address barriers to access while respecting sociocultural norms. Ensuring that fish is readily available, affordable, and accessible to all, especially children, without disrupting existing cultural practices, is essential for maximizing the nutritional benefits of fish.

## Author contributions

The authors’ responsibilities were as follows – RKR: conceptualized the study, conducted the data analysis, drafted, revised, and finalized the manuscript; SB, BB, BCR, RK, SKD, WNG-W, EHA, CMR, APP, SVS, CDG: reviewed or/and provided feedback on the drafts and revisions of the manuscript; EHA, CMR, APP, SVS, CDG: provided overall oversight and guidance of the study execution; and all authors: had full access to entire datasets used in the study and collectively assumed final responsibility for the decision to submit the manuscript for publication.

## Data availability

Data from the National Family Health Surveys, as detailed in the manuscript, can be obtained by submitting a request through the DHS Program's official website: https://dhsprogram.com/.

## Funding

This research was conducted as part of the Consortium of International Agricultural Research Centers (CGIAR) Research Initiative on Aquatic Foods with funding from the CGIAR Trust Fund donors: https://www.cgiar.org/funders/. The funders had no role in the study design, analysis or interpretation of the data, preparation of the manuscript, or decision to publish.

## Conflict of interest

The authors declare no competing interests.
