# Supplementary material for: Relationship between Fish Consumption and Undernutrition among Young Indian Children
Source: Curr Dev Nutr. 2025 Nov 29;10(1):107610. doi: 10.1016/j.cdnut.2025.107610 (PMC12799933; doi:10.1016/j.cdnut.2025.107610)
Supplement: Multimedia component 1 [file mmc1.pdf]

Relationship between fish consumption and undernutrition among young Indian children

Rajesh Kumar Rai[1,2,3,4,*], Sabri Bromage[5,6], Baban Bayan[7], Baishnaba Charan Ratha[7], Rockli Kim[8], Sourabh Kumar Dubey[7], Wanjiku N Gichohi-Wainaina[9], Edward H Allison[9], Cristiano M Rossignoli[9], Arun Panemangalore Padiyar[7], S V Subramanian[10,11], Christopher D Golden[4,6,12]

[1] Birbhum Population Project, Society for Health and Demographic Surveillance, Suri, West Bengal, India; [2] School of Public Health, San Diego State University, San Diego, California, USA; [3] Human Nutrition Unit, Institute of Nutrition, Mahidol University, Phutthamonthon, Nakhon Pathom, Thailand; [4] Department of Global Health and Population, Harvard T H Chan School of Public Health, Harvard University, Boston, Massachusetts, USA; [5] Community Nutrition Unit, Institute of Nutrition, Mahidol University, Phutthamonthon, Nakhon Pathom, Thailand; [6] Department of Nutrition, Harvard T H Chan School of Public Health, Harvard University, Boston, Massachusetts, USA; [7] WorldFish, New Delhi, India; [8] Division of Health Policy & Management, College of Health Science, Korea University, Seoul, Korea; [9] WorldFish, Jalan Batu Maung, Penang, Malaysia; [10] Harvard Center for Population and Development Studies, Harvard University, Cambridge, Massachusetts, USA; [11] Department of Social and Behavioral Sciences, Harvard T H Chan School of Public Health, Harvard University, Boston, Massachusetts, USA; [12] Department of Environmental Health, Harvard T H Chan School of Public Health, Harvard University, Boston, Massachusetts, USA.

*Corresponding author. *E-mail address:* rajesh.iips28@gmail.com (R. K. Rai).

Online supplement

Table S1. Percentage of children reported consuming any fresh or dried fish or shellfish during the day prior to survey date, by state/ union territory, NFHS 2005-06, 2015-16, and 2019-21.

State/ union territory	NFHS 2005-06 % (95% CI)	NFHS 2015-16 % (95% CI)	NFHS 2019-21 % (95% CI)
Andhra Pradesh (including Telangana) ¥	1.9 (1.4, 2.5)	2.5 (2.2, 2.8)	2.2 (1.9, 2.6)
Arunachal Pradesh	6.9 (2.5, 18.0)	14.1 (8.7, 21.9)	13.0 (7.3, 22.2)
Assam	10.1 (8.4, 12.1)	6.3 (5.5, 7.2)	4.0 (3.3, 4.8)
Bihar	1.6 (1.3, 2.0)	3.3 (3.1, 3.6)	6.0 (5.6, 6.3)
Chhattisgarh	3.7 (2.6, 5.1)	1.4 (1.1, 1.8)	3.5 (2.9, 4.2)
Delhi	1.6 (0.7, 3.4)	1.8 (1.2, 2.6)	2.3 (1.6, 3.2)
Goa	30.8 (18.6, 46.6)	10.5 (5.9, 17.9)	16.1 (10.1, 24.8)
Gujarat	1.8 (1.3, 2.4)	1.0 (0.8, 1.3)	2.0 (1.7, 2.4)
Haryana	0.6 (0.2, 1.5)	0.6 (0.4, 0.9)	1.2 (0.8, 1.7)
Himachal Pradesh	5.2 (2.8, 9.5)	0.5 (0.1, 1.5)	0.4 (0.1, 1.5)
Jammu & Kashmir (including Ladakh) ±	1.0 (0.4, 2.8)	6.2 (4.9, 7.7)	9.5 (7.6, 11.7)
Jharkhand	2.6 (1.9, 3.6)	2.2 (1.8, 2.7)	6.2 (5.5, 6.9)
Karnataka	6.1 (5.2, 7.2)	5.0 (4.5, 5.6)	7.7 (7.0, 8.4)
Kerala	36.0 (32.6, 39.5)	15.0 (13.5, 16.6)	27.0 (25.1, 28.9)
Madhya Pradesh	1.2 (0.8, 1.6)	1.4 (1.2, 1.6)	2.0 (1.8, 2.4)
Maharashtra	1.0 (0.7, 1.4)	3.1 (2.8, 3.4)	2.4 (2.1, 2.7)
Manipur	26.8 (18.8, 36.7)	20.7 (16.2, 26.0)	22.6 (17.6, 28.6)
Meghalaya	11.0 (6.9, 17.2)	15.9 (12.8, 19.5)	20.8 (17.5, 24.6)
Mizoram	5.5 (1.4, 18.7)	19.6 (13.3, 27.9)	9.9 (5.3, 17.7)
Nagaland	3.4 (1.0, 10.5)	11.9 (8.2, 17.1)	8.7 (4.8, 15.3)
Odisha	5.4 (4.4, 6.7)	3.9 (3.3, 4.5)	6.0 (5.2, 6.8)
Punjab	1.5 (0.8, 2.6)	0.5 (0.3, 1.0)	0.5 (0.3, 0.9)
Rajasthan	0.3 (0.1, 0.5)	0.2 (0.1, 0.3)	0.4 (0.3, 0.5)
Sikkim	4.1 (0.4, 30.0)	11.2 (3.8, 28.8)	14.9 (5.1, 36.2)
Tamil Nadu	6.2 (5.1, 7.6)	22.5 (21.6, 23.5)	9.8 (9.1, 10.6)
Tripura	24.4 (17.4, 33.1)	4.6 (2.7, 8.0)	2.6 (1.3, 5.4)
Uttar Pradesh	0.9 (0.7, 1.1)	0.7 (0.6, 0.8)	1.6 (1.5, 1.8)
Uttarakhand	3.7 (2.2, 6.4)	0.8 (0.4, 1.5)	0.9 (0.5, 1.8)
West Bengal	23.1 (21.7, 24.7)	8.6 (8.1, 9.3)	11.0 (10.3, 11.7)
Andaman & Nicobar Islands	dnc	18.0 (7.4, 37.6)	14.7 (4.6, 38.4)
Andhra Pradesh¥	nr	2.0 (1.6, 2.4)	2.3 (1.9, 2.8)
Chandigarh	dnc	nc	1.4 (0.2, 10.8)
Dadra & Nagar Haveli	dnc	nc	2.9 (0.4, 19.2)
Daman & Diu	dnc	6.2 (0.8, 35.3)	5.4 (0.3, 51.6)
Jammu & Kashmir±	nr	6.2 (4.9, 7.7)	9.5 (7.6, 11.8)
Ladakh±	nr	3.9 (0.4, 31.8)	7.9 (1.0, 41.6)
Lakshadweep	dnc	24.8 (5.2, 66.6)	28.1 (4.6, 76.0)
Puducherry	dnc	28.2 (20.2, 38.0)	18.6 (11.4, 28.8)
Telangana¥	nr	3.1 (2.6, 3.7)	2.0 (1.5, 2.6)
Total	4.4 (4.2, 4.6)	3.9 (3.8, 4.0)	4.5 (4.4, 4.6)

¥ Separate estimates for Andhra Pradesh and Telangana were not available in NFHS 2005-2006. However, separate data were collected for these two states in NFHS 2015-2016 and NFHS 2019-2021.

± Ladakh was an integral part of Jammu & Kashmir during NFHS 2005-2006. However, in NFHS 2015-2016 and NFHS 2019-2021, district-level data were collected, allowing for separate estimates for Ladakh and Jammu & Kashmir. This change coincided with the establishment of Ladakh as a Union Territory in 2019.

CI: confidence interval, dnc: data not collected in 2005-06; NFHS: National Family Health Survey, nc: no case, nr: not required

Table S2: Percentage of children consuming fresh or dried Fish or shellfish the day prior to survey, by state/union territory, NFHS 2019-21, and district range or DR (minimum-maximum) therein, for age group 0-59 Months.

State/ union territory	Number of districts	DR (0-59 months)
Andaman & Nicobar Islands	3	6.6-21.6
Andhra Pradesh	13	0.0-8.9
Arunachal Pradesh	20	4.1-20.0
Assam	33	0.0-10.5
Bihar	38	1.3-13.0
Chandigarh	1	1.4
Chhattisgarh	27	0.0-10.4
Dadra & Nagar Haveli and Daman & Diu	3	1.4-23.1
Delhi	11	0.0-6.7
Goa	2	15.8-16.6
Gujarat	33	0.0-12.2
Haryana	22	0.0-4.4
Himachal Pradesh	12	0.0- 2.7
Jammu & Kashmir	20	0.0- 20.2
Jharkhand	24	1.1-12.3
Karnataka	30	1.8-17.9
Kerala	14	11.1-38.4
Ladakh	2	4.0-11.9
Lakshadweep	1	28.1
Madhya Pradesh	51	0.0-8.9
Maharashtra	36	0.0-5.6
Manipur	9	11.2-38.3
Meghalaya	11	12.1-38.6
Mizoram	8	0.9-15.9
Nagaland	11	1.7-16.7
Odisha	30	0.63-11.1
Puducherry	4	5.6-28.1
Punjab	22	0.0-2.9
Rajasthan	33	0.0-1.4
Sikkim	4	7.7-19.9
Tamil Nadu	32	0.0-34.6
Telangana	31	0.0-5.9
Tripura	8	0.0-6.7
Uttar Pradesh	75	0.0-7.1
Uttarakhand	13	0.0-3.4
West Bengal	20	1.2-20.6
India	707	0.0 – 38.6

NFHS: National Family Health Survey

Table S3. Prevalence (%) of stunting and wasting among NFC and FC groups of children (aged 6-23 months) by select background characteristics.

	Stunting				Wasting			
	NFC	FC	NFC	FC	NFC	FC	NFC	FC
	n	n	% (95% CI)	% (95% CI)	n	n	% (95% CI)	% (95% CI)
**Age of child (months)**								
6-11	19,042	585	24.6 (24.0, 25.2)	24.7 (21.2, 28.5)	18,863	585	23.3 (22.7, 23.9)	20.2 (17.0, 23.9)
12-23	36,761	3,172	39.8 (39.3, 40.3)	38.9 (37.1, 40.6)	36,551	3,146	19.6 (19.2, 20.0)	19.9 (18.5, 21.4)
**Sex of child**								
Male	28,779	1,917	36.9 (36.3, 37.5)	40.9 (38.7, 43.2)	28,550	1,913	21.9 (21.4, 22.4)	20.6 (18.8, 22.5)
Female	27,024	1,840	32.1 (31.6, 32.7)	31.9 (29.7, 34.3)	26,864	1,818	19.7 (19.3, 20.2)	19.2 (17.3, 21.2)
**Birth order**								
1	22,058	1,288	31.5 (30.9, 32.1)	33.3 (30.9, 35.9)	21,942	1,273	19.4 (18.9, 19.9)	20.6 (18.5, 22.9)
2	18,540	1,309	34.2 (33.6, 34.9)	35.0 (32.4, 37.6)	18,345	1,295	20.5 (20.0, 21.1)	16.1 (14.1, 18.2)
3	8,503	543	37.2 (36.2, 38.3)	40.1 (35.6, 44.7)	8,452	547	22.8 (21.9, 23.7)	24.2 (20.5, 28.4)
≥4	6,702	617	43.3 (42.1, 44.6)	50.1 (45.0, 55.1)	6,675	616	24.5 (23.4, 25.6)	25.0 (20.9, 29.7)
**Consumed animal product (excluding fish)**								
No	40,327	423	34.5 (34.0, 34.9)	35.0 (30.7, 39.7)	40,065	416	21.6 (21.2, 22.0)	16.9 (13.6, 20.7)
Yes	15,476	3,334	34.9 (34.2, 35.7)	36.9 (35.2, 38.6)	15,349	3,315	19.0 (18.4, 19.6)	20.4 (19.0, 21.8)
**Consumed carbohydrate rich food**								
No	21,936	302	33.2 (32.6, 33.9)	35.7 (30.3, 41.4)	21,810	295	22.0 (21.4, 22.5)	23.8 (19.2, 29.2)
Yes	33,867	3,455	35.5 (35.0, 36.0)	36.7 (35.1, 38.4)	33,604	3,436	20.1 (19.7, 20.6)	19.6 (18.2, 21.0)
**Consumed pumpkin, carrots, Squash (yellow or orange inside)**								
No	44,282	1,236	34.2 (33.8, 34.7)	36.7 (34.1, 39.4)	43,946	1,230	21.2 (20.9, 21.6)	15.5 (13.6, 17.6)
Yes	11,521	2,521	36.0 (35.1, 36.9)	36.6 (34.6, 38.7)	11,468	2,501	19.4 (18.6, 20.1)	22.4 (20.7, 24.2)
**Consumed dark leafy vegetables**								
No	38,911	817	33.4 (33.0, 33.9)	37.0 (33.8, 40.3)	38,673	806	21.2 (20.8, 21.6)	17.3 (14.8, 20.0)
Yes	16,892	2,940	37.5 (36.7, 38.2)	36.6 (34.7, 38.4)	16,741	2,925	20.1 (19.5, 20.7)	20.8 (19.2, 22.4)
**Consumed Fruits**								
No	39,427	654	34.4 (33.9, 34.9)	42.8 (38.9, 46.7)	39,174	648	21.3 (20.9, 21.7)	18.7 (15.8, 22.0)
Yes	16,376	3,103	35.1 (34.4, 35.8)	35.3 (33.6, 37.1)	16,240	3,083	19.7 (19.1, 20.3)	20.2 (18.7, 21.7)
**Consumed lentils or solid/ semi-solid food**								

No	39,492	627	34.6 (34.2, 35.1)	39.2 (35.4, 43.1)	39,192	627	21.5 (21.1, 21.9)	19.5 (16.5, 22.8)
Yes	16,311	3,130	34.5 (33.7, 35.2)	36.1 (34.3, 37.9)	16,222	3,104	19.3 (18.7, 19.9)	20.0 (18.6, 21.6)
**ARI**								
No	51,344	3,399	34.6 (34.2, 35.0)	35.2 (33.5, 36.9)	50,978	3,373	20.7 (20.4, 21.1)	19.4 (18.0, 20.8)
Yes	4,459	358	34.7 (33.4, 36.0)	47.7 (42.8, 52.6)	4,436	358	22.5 (21.3, 23.7)	24.2 (20.3, 28.7)
**Diarrhea**								
No	50,134	3,370	34.5 (34.1, 34.9)	36.0 (34.4, 37.8)	49,761	3,340	20.5 (20.2, 20.9)	19.8 (18.4, 21.3)
Yes	5,669	387	35.6 (34.4, 36.8)	41.3 (36.6, 46.2)	5,653	391	23.7 (22.6, 24.8)	20.9 (17.2, 25.2)
**Had low birthweight**								
No	42,394	2,904	32.1 (31.7, 32.6)	34.3 (32.5, 36.2)	42,052	2,880	19.4 (19.0, 19.8)	17.9 (16.5, 19.4)
Yes	9,117	484	42.7 (41.7, 43.7)	44.8 (40.7, 49.0)	9,080	480	25.3 (24.5, 26.2)	24.6 (21.2, 28.3)
Missing	4,292	369	41.3 (39.8, 42.8)	43.4 (37.2, 49.9)	4,282	371	25.6 (24.2, 27.0)	31.9 (26.2, 38.2)
**Household size**								
1-4	14,278	1,097	34.2 (33.5, 35.0)	32.8 (29.9, 35.8)	14,161	1,092	21.5 (20.8, 22.2)	18.2 (15.9, 20.7)
5-6	20,795	1,508	34.6 (33.9, 35.2)	34.9 (32.5, 37.4)	20,652	1,497	20.3 (19.8, 20.9)	21.7 (19.7, 24.0)
≥7	20,730	1,152	34.8 (34.2, 35.5)	42.6 (39.7, 45.7)	20,601	1,142	20.9 (20.4, 21.5)	19.1 (16.8, 21.6)
**Religion**								
Hinduism	42,048	2,054	34.4 (33.9, 34.8)	36.1 (34.2, 38.0)	41,773	2,033	21.1 (20.7, 21.5)	20.7 (19.1, 22.3)
Islam	7,590	680	36.6 (35.6, 37.6)	41.0 (37.6, 44.5)	7,490	675	20.7 (19.9, 21.6)	17.6 (15.1, 20.4)
Others	6,165	1,023	31.6 (29.7, 33.5)	28.7 (23.5, 34.5)	6,151	1,023	17.1 (15.6, 18.7)	20.3 (15.8, 25.6)
**Social group**								
SC	11,593	625	38.1 (37.3, 38.9)	39.4 (36.0, 42.9)	11,523	620	21.8 (21.1, 22.5)	21.1 (18.3, 24.2)
ST	10,740	1,280	39.3 (38.0, 40.6)	42.8 (37.9, 47.8)	10,646	1,276	25.7 (24.5, 26.9)	26.9 (22.7, 31.6)
OBC	21,765	1,134	33.8 (33.2, 34.4)	36.6 (34.1, 39.1)	21,598	1,117	20.5 (20.0, 21.0)	18.5 (16.6, 20.6)
Others	11,705	718	30.6 (29.8, 31.4)	31.7 (28.7, 34.8)	11,647	718	18.6 (17.9, 19.3)	18.1 (15.7, 20.8)
**Wealth index**								
Poorest	14,525	1,185	43.7 (42.8, 44.5)	46.9 (43.7, 50.1)	14,458	1,180	26.6 (25.9, 27.4)	26.9 (24.2, 29.8)
Poorer	13,021	857	39.0 (38.1, 39.8)	39.3 (35.7, 43.0)	12,934	861	21.8 (21.1, 22.5)	18.2 (15.5, 21.2)
Middle	11,170	678	33.2 (32.3, 34.0)	34.3 (30.8, 38.1)	11,076	674	19.7 (19.0, 20.4)	19.8 (16.9, 23.0)
Richer	9,522	629	28.6 (27.8, 29.5)	30.6 (27.2, 34.2)	9,445	617	18.3 (17.5, 19.0)	15.2 (12.6, 18.1)
Richest	7,565	408	23.6 (22.8, 24.5)	24.8 (21.2, 28.8)	7,501	399	15.4 (14.7, 16.2)	15.5 (12.5, 18.9)
**Locality of residence**								
Urban	10,981	801	30.1 (29.4, 30.9)	29.8 (27.0, 32.7)	10,879	784	18.8 (18.2, 19.5)	18.6 (16.3, 21.2)
Rural	44,822	2,956	36.1 (35.7, 36.6)	39.4 (37.5, 41.3)	44,535	2,947	21.6 (21.2, 22.0)	20.4 (18.9, 22.1)
**Regional fish availability**								
Marine water fish	12,197	1,125	33.0 (32.3, 33.7)	32.8 (30.4, 35.3)	12,077	1,103	21.3 (20.7, 21.9)	16.9 (15.0, 19.0)
Freshwater fish	32,230	2,347	36.8 (36.3, 37.4)	39.7 (37.5, 42.0)	32,028	2,346	21.8 (21.3, 22.2)	22.1 (20.3, 24.0)
Others	11,376	285	29.4 (28.4, 30.4)	34.7 (27.2, 43.2)	11,309	282	16.6 (15.8, 17.4)	20.8 (14.7, 28.6)
**Age of mother (in years)**								

15-19	2,230	140	36.7 (34.9, 38.7)	43.3 (36.3, 50.6)	2,212	139	23.4 (21.7, 25.1)	14.3 (9.9, 20.2)
20-24	20,751	1,207	35.5 (34.8, 36.1)	38.7 (36.1, 41.4)	20,612	1,192	20.4 (19.8, 20.9)	19.8 (17.7, 22.1)
25-29	20,620	1,337	33.8 (33.1, 34.4)	35.1 (32.5, 37.8)	20,457	1,326	21.2 (20.6, 21.8)	20.1 (17.9, 22.4)
30-34	8,628	694	34.0 (32.9, 35.0)	34.1 (30.2, 38.2)	8,577	701	20.1 (19.2, 21.0)	20.9 (17.7, 24.5)
35-49	3,574	379	33.8 (32.1, 35.5)	34.0 (28.0, 40.5)	3,556	373	22.3 (20.8, 23.8)	22.0 (17.0, 28.1)
**Educational attainment**								
No education	10,781	696	43.8 (42.9, 44.8)	47.3 (43.2, 51.4)	10,685	696	25.7 (24.9, 26.6)	30.1 (26.5, 34.0)
Incomplete primary	6,600	453	39.7 (38.5, 40.9)	39.0 (34.0, 44.3)	6,571	442	21.6 (20.6, 22.6)	22.8 (18.7, 27.6)
Incomplete secondary	28,738	1,944	33.5 (32.9, 34.0)	38.1 (35.9, 40.4)	28,576	1,939	20.4 (19.9, 20.9)	17.4 (15.7, 19.3)
Complete secondary	1,169	75	31.5 (29.0, 34.2)	39.7 (29.5, 50.9)	1,152	73	20.8 (18.6, 23.2)	23.6 (15.5, 34.3)
Higher	8,515	589	24.7 (23.8, 25.5)	22.5 (19.5, 25.8)	8,430	581	16.3 (15.6, 17.1)	15.9 (13.3, 18.8)
**Maternal BMI**								
Underweight	12,529	687	40.9 (40.0, 41.7)	43.5 (39.9, 47.1)	12,435	684	26.8 (26.0, 27.6)	24.9 (21.9, 28.2)
Normal	27,844	1,885	35.1 (34.6, 35.7)	37.7 (35.4, 40.2)	27,656	1,870	20.8 (20.3, 21.3)	20.2 (18.3, 22.3)
Overweight/obesity	15,430	1,185	28.5 (27.8, 29.2)	30.8 (28.2, 33.5)	15,323	1,177	16.0 (15.5, 16.6)	16.3 (14.3, 18.6)
**Tobacco user**								
No	52,804	3,336	34.3 (33.9, 34.7)	36.6 (34.9, 38.2)	52,429	3,321	20.7 (20.4, 21.1)	19.7 (18.4, 21.1)
Yes	2,999	421	44.1 (41.6, 46.6)	39.4 (31.2, 48.2)	2,985	410	25.3 (23.2, 27.6)	24.7 (17.9, 33.1)
**Alcohol user**								
No	55,078	3,680	34.6 (34.2, 35.0)	36.6 (35.0, 38.2)	54,711	3,651	20.8 (20.5, 21.2)	19.9 (18.6, 21.3)
Yes	725	77	41.7 (35.9, 47.8)	47.7 (30.0, 66.0)	703	80	28.5 (23.3, 34.4)	25.9 (12.6, 45.8)
**Hypertensive**								
No	46,480	3,059	34.7 (34.3, 35.2)	35.9 (34.2, 37.7)	46,147	3,029	20.8 (20.5, 21.2)	19.5 (18.0, 21.0)
Yes	5,906	396	32.9 (31.7, 34.1)	45.6 (40.1, 51.2)	5,851	395	19.8 (18.8, 20.8)	18.1 (14.2, 22.8)
Missing	3,417	302	35.8 (34.1, 37.5)	34.3 (29.0, 40.0)	3,416	307	23.0 (21.6, 24.5)	26.4 (21.6, 31.8)
**Hyperglycemic**								
No	54,281	3,630	34.8 (34.4, 35.2)	36.7 (35.1, 38.4)	53,906	3,604	20.9 (20.6, 21.3)	20.0 (18.6, 21.4)
Yes	970	76	28.1 (25.4, 30.9)	29.3 (20.5, 40.0)	965	77	20.5 (18.1, 23.0)	16.5 (10.0, 26.0)
Missing	552	51	31.6 (28.2, 35.3)	46.5 (33.0, 60.5)	543	50	16.7 (14.0, 19.8)	22.1 (12.3, 36.5)
**Had ≥4 ANC visits**								
No	22,628	1,517	36.5 (35.9, 37.2)	41.1 (38.4, 43.8)	22,537	1,518	22.2 (21.7, 22.8)	25.4 (23.1, 27.9)
Yes	30,715	2,177	32.5 (31.9, 33.0)	33.9 (31.8, 35.9)	30,415	2,153	20.3 (19.8, 20.7)	16.4 (14.9, 18.1)
Missing	2,460	63	43.6 (41.7, 45.6)	38.4 (25.2, 53.6)	2,462	60	16.0 (14.6, 17.5)	23.1 (12.6, 38.4)
**Consumed ≥ 100 IFA**								
No	30,307	1,965	36.1 (35.6, 36.7)	39.6 (37.3, 42.1)	30,083	1,950	22.1 (21.6, 22.6)	22.0 (20.0, 24.2)
Yes	23,036	1,729	31.6 (31.1, 32.2)	34.0 (31.9, 36.2)	22,869	1,721	19.8 (19.3, 20.3)	18.0 (16.3, 19.9)
Missing	2,460	63	43.6 (41.7, 45.6)	38.4 (25.2, 53.6)	2,462	60	16.0 (14.6, 17.5)	23.1 (12.6, 38.4)
**Caesarean section delivery**								

No	43,936	2,911	36.5 (36.0, 36.9)	39.8 (37.9, 41.7)	43,646	2,894	21.6 (21.2, 22.0)	22.7 (21.1, 24.5)
Yes	11,867	846	28.4 (27.7, 29.2)	28.8 (26.0, 31.7)	11,768	837	18.3 (17.7, 19.0)	12.7 (10.7, 14.9)
**Total**	55,803	3,757	34.6 (34.2, 35.0)	36.7 (35.1, 38.3)	55,414	3,731	20.9 (20.5, 21.2)	19.9 (18.6, 21.3)

All n are unweighted.

ANC: antenatal care, ARI: acute respiratory infection, BMI: body mass index, CI: confidence interval, IFA: iron-and-folic-acid, n: sample, OBCs: other backward classes, SCs: scheduled castes, STs: scheduled tribes

FC or children who consumed fish; NFC or children who did not consume fish.

Table S4. Prevalence (%) of underweight and any anthropometric failure among NFC and FC groups of children (aged 6-23 months) by select background characteristics.

	Underweight				Any anthropometric failure			
	NFC	FC	NFC	FC	NFC	FC	NFC	FC
	n	n	% (95% CI)	% (95% CI)	n	n	% (95% CI)	% (95% CI)
**Age of child (months)**								
6-11	19,772	607	26.3 (25.7, 26.9)	26.4 (22.9, 30.3)	19,388	595	48.9 (48.2, 49.6)	45.9 (41.7, 50.1)
12-23	37,819	3,252	31.2 (30.7, 31.6)	30.2 (28.5, 31.8)	37,255	3,202	56.6 (56.1, 57.1)	54.8 (53.0, 56.6)
**Sex of child**								
Male	29,775	1,965	31.8 (31.2, 32.3)	33.3 (31.2, 35.5)	29,302	1,940	56.7 (56.1, 57.2)	57.6 (55.3, 59.9)
Female	27,816	1,894	27.0 (26.5, 27.5)	25.5 (23.4, 27.6)	27,341	1,857	51.1 (50.5, 51.7)	48.8 (46.4, 51.2)
**Birth order**								
1	22,797	1,314	25.8 (25.2, 26.4)	25.6 (23.3, 28.0)	22,389	1,299	50.1 (49.4, 50.7)	48.7 (46.1, 51.4)
2	19,093	1,348	28.6 (28.0, 29.3)	26.9 (24.6, 29.4)	18,791	1,318	53.4 (52.7, 54.1)	50.6 (47.9, 53.3)
3	8,780	561	34.3 (33.3, 35.3)	36.9 (32.6, 41.3)	8,641	553	58.1 (57.0, 59.1)	60.7 (56.1, 65.0)
≥4	6,921	636	38.8 (37.6, 40.0)	44.0 (39.1, 49.0)	6,822	627	64.1 (62.9, 65.3)	70.3 (65.5, 74.7)
**Consumed animal product (excluding fish)**								
No	41,647	436	29.9 (29.5, 30.4)	26.1 (22.2, 30.4)	40,983	426	54.4 (53.9, 54.8)	50.9 (46.2, 55.6)
Yes	15,944	3,423	28.3 (27.6, 29.0)	30.1 (28.5, 31.7)	15,660	3,371	52.9 (52.1, 53.7)	53.8 (52.0, 55.5)
**Consumed carbohydrate rich food**								
No	22,769	310	29.3 (28.7, 29.9)	26.3 (21.6, 31.7)	22,377	305	54.4 (53.7, 55.0)	53.1 (47.3, 58.8)
Yes	34,822	3,549	29.6 (29.1, 30.0)	29.9 (28.3, 31.5)	34,266	3,492	53.7 (53.2, 54.2)	53.4 (51.7, 55.2)
**Consumed pumpkin, carrots, Squash (yellow or orange inside)**								
No	45,739	1,270	29.5 (29.1, 29.9)	26.5 (24.1, 29.0)	44,960	1,250	54.0 (53.5, 54.4)	51.2 (48.4, 53.9)
Yes	11,852	2,589	29.3 (28.5, 30.1)	31.3 (29.4, 33.3)	11,683	2,547	54.0 (53.1, 54.9)	54.7 (52.6, 56.7)
**Consumed dark leafy vegetables**								
No	40,218	831	29.2 (28.7, 29.6)	26.7 (23.8, 29.8)	39,559	825	53.3 (52.8, 53.8)	51.6 (48.2, 55.0)
Yes	17,373	3,028	30.2 (29.5, 30.9)	30.5 (28.8, 32.3)	17,084	2,972	55.6 (54.8, 56.3)	54.0 (52.1, 55.9)
**Consumed Fruits**								
No	40,727	673	29.8 (29.4, 30.3)	30.7 (27.2, 34.5)	40,086	662	54.4 (53.9, 54.8)	58.0 (54.0, 61.8)
Yes	16,864	3,186	28.6 (27.9, 29.3)	29.3 (27.7, 31.0)	16,557	3,135	53.0 (52.2, 53.7)	52.4 (50.6, 54.2)
**Consumed lentils or solid/ semi-solid food**								

No	40,783	645	29.8 (29.3, 30.2)	32.0 (28.5, 35.7)	40,120	641	54.4 (53.9, 54.8)	56.3 (52.4, 60.2)
Yes	16,808	3,214	28.8 (28.1, 29.5)	29.1 (27.4, 30.7)	16,523	3,156	53.0 (52.2, 53.8)	52.7 (50.9, 54.6)
**ARI**								
No	52,981	3,494	29.3 (28.9, 29.7)	28.3 (26.7, 29.9)	52,120	3,433	53.9 (53.4, 54.3)	52.9 (51.1, 54.6)
Yes	4,610	365	31.0 (29.7, 32.3)	39.7 (35.0, 44.6)	4,523	364	55.2 (53.8, 56.6)	57.4 (52.5, 62.1)
**Diarrhea**								
No	51,725	3,459	29.0 (28.6, 29.4)	29.0 (27.5, 30.6)	50,876	3,405	53.6 (53.2, 54.1)	53.0 (51.2, 54.7)
Yes	5,866	400	33.4 (32.3, 34.6)	33.9 (29.4, 38.6)	5,767	392	56.6 (55.3, 57.8)	56.7 (51.8, 61.4)
**Had low birthweight**								
No	43,741	2,986	26.3 (25.9, 26.7)	26.0 (24.4, 27.7)	42,967	2,933	51.0 (50.5, 51.5)	50.9 (49.0, 52.8)
Yes	9,418	495	40.0 (39.1, 41.0)	38.9 (35.0, 43.0)	9,309	490	63.4 (62.4, 64.3)	59.3 (55.2, 63.3)
Missing	4,432	378	37.7 (36.3, 39.2)	47.7 (41.4, 54.0)	4,367	374	62.6 (61.1, 64.1)	67.4 (61.2, 73.1)
**Household size**								
1-4	14,732	1,123	29.2 (28.4, 29.9)	25.7 (23.1, 28.5)	14,474	1,108	53.9 (53.1, 54.7)	50.2 (47.0, 53.3)
5-6	21,499	1,558	29.5 (28.9, 30.1)	31.1 (28.8, 33.5)	21,130	1,532	53.7 (53.0, 54.4)	52.6 (50.0, 55.2)
≥7	21,360	1,178	29.6 (29.0, 30.2)	31.2 (28.5, 34.0)	21,039	1,157	54.3 (53.6, 54.9)	57.6 (54.6, 60.6)
**Religion**								
Hinduism	43,381	2,110	29.6 (29.2, 30.0)	30.8 (29.0, 32.7)	42,681	2,076	53.8 (53.3, 54.2)	52.5 (50.5, 54.5)
Islam	7,868	696	29.9 (29.0, 30.9)	28.1 (25.1, 31.3)	7,733	688	56.7 (55.6, 57.7)	56.8 (53.3, 60.2)
Others	6,342	1,053	25.2 (23.5, 27.0)	22.8 (18.1, 28.2)	6,229	1,033	47.6 (45.6, 49.7)	51.1 (45.1, 57.1)
**Social group**								
SC	11,930	644	32.7 (31.9, 33.5)	37.5 (34.1, 40.9)	11,742	634	57.1 (56.3, 58.0)	58.0 (54.5, 61.5)
ST	11,109	1,316	38.6 (37.3, 39.8)	32.1 (27.6, 36.8)	10,929	1,293	61.8 (60.5, 63.1)	61.7 (56.8, 66.4)
OBC	22,429	1,158	28.4 (27.8, 29.0)	28.2 (25.9, 30.6)	22,089	1,140	53.2 (52.6, 53.8)	51.8 (49.2, 54.4)
Others	12,123	741	24.3 (23.5, 25.0)	23.9 (21.2, 26.8)	11,883	730	48.8 (47.9, 49.7)	48.4 (45.1, 51.6)
**Wealth index**								
Poorest	15,014	1,215	41.9 (41.1, 42.7)	42.8 (39.7, 45.9)	14,823	1,203	65.9 (65.1, 66.7)	65.8 (62.8, 68.8)
Poorer	13,434	887	33.1 (32.3, 33.9)	28.5 (25.3, 32.0)	13,224	871	58.7 (57.8, 59.5)	55.1 (51.3, 58.7)
Middle	11,504	694	27.1 (26.3, 27.9)	26.3 (23.1, 29.8)	11,319	684	52.1 (51.2, 53.0)	50.0 (46.2, 53.9)
Richer	9,836	643	22.6 (21.8, 23.4)	21.1 (18.2, 24.3)	9,634	630	47.1 (46.2, 48.1)	46.7 (42.9, 50.5)
Richest	7,803	420	16.9 (16.1, 17.7)	21.7 (18.4, 25.5)	7,643	409	39.6 (38.6, 40.6)	40.7 (36.5, 45.1)
**Locality of residence**								
Urban	11,363	816	24.6 (23.9, 25.3)	23.5 (21.0, 26.2)	11,126	800	49.0 (48.1, 49.8)	46.1 (43.0, 49.3)
Rural	46,228	3,043	31.2 (30.7, 31.6)	32.0 (30.2, 33.8)	45,517	2,997	55.7 (55.2, 56.2)	56.3 (54.3, 58.2)
**Regional fish availability**								
Marine water fish	12,609	1,152	28.1 (27.5, 28.8)	24.9 (22.8, 27.2)	12,375	1,128	53.0 (52.3, 53.8)	49.6 (47.0, 52.2)
Freshwater fish	33,220	2,409	32.0 (31.5, 32.5)	33.5 (31.4, 35.7)	32,740	2,377	56.6 (56.1, 57.2)	56.2 (53.9, 58.4)
Others	11,762	298	22.6 (21.7, 23.5)	24.1 (17.7, 31.8)	11,528	292	45.8 (44.8, 46.9)	54.7 (46.4, 62.9)
**Age of mother (in years)**								

15-19	2,309	145	34.6 (32.7, 36.4)	36.6 (30.1, 43.7)	2,274	141	59.3 (57.4, 61.2)	52.6 (45.4, 59.8)
20-24	21,371	1,236	29.7 (29.1, 30.3)	27.5 (25.2, 30.0)	21,048	1,216	54.2 (53.6, 54.9)	53.9 (51.2, 56.6)
25-29	21,284	1,368	28.7 (28.1, 29.3)	30.7 (28.2, 33.3)	20,915	1,352	53.3 (52.6, 53.9)	52.7 (49.9, 55.5)
30-34	8,922	720	29.0 (28.0, 30.0)	29.0 (25.3, 32.9)	8,776	706	52.9 (51.8, 54.0)	52.5 (48.3, 56.6)
35-49	3,705	390	30.2 (28.6, 31.8)	31.1 (25.4, 37.4)	3,630	382	55.1 (53.3, 56.9)	56.9 (50.3, 63.2)
**Educational attainment**								
No education	11,114	719	40.6 (39.7, 41.6)	42.9 (38.9, 47.0)	10,998	712	65.3 (64.4, 66.2)	70.3 (66.5, 73.9)
Incomplete primary	6,803	464	34.5 (33.3, 35.7)	38.5 (33.6, 43.7)	6,696	455	59.2 (57.9, 60.4)	59.8 (54.5, 64.8)
Incomplete secondary	29,709	2,000	28.1 (27.6, 28.6)	27.4 (25.4, 29.5)	29,175	1,968	52.8 (52.2, 53.4)	52.4 (50.1, 54.8)
Complete secondary	1,204	80	24.9 (22.6, 27.4)	24.3 (16.2, 34.7)	1,176	73	51.2 (48.4, 54.1)	49.3 (38.3, 60.3)
Higher	8,761	596	18.5 (17.8, 19.3)	20.0 (17.2, 23.2)	8,598	589	41.7 (40.7, 42.6)	38.8 (35.2, 42.5)
**Maternal BMI**								
Underweight	12,886	709	40.5 (39.7, 41.3)	40.0 (36.5, 43.5)	12,730	699	63.9 (63.0, 64.7)	61.8 (58.2, 65.2)
Normal	28,768	1,932	29.5 (29.0, 30.1)	30.2 (28.0, 32.5)	28,292	1,906	54.7 (54.1, 55.3)	55.1 (52.6, 57.5)
Overweight/obesity	15,937	1,218	20.2 (19.6, 20.9)	22.1 (19.8, 24.5)	15,621	1,192	44.4 (43.7, 45.2)	45.7 (42.8, 48.6)
**Tobacco user**								
No	54,500	3,429	29.2 (28.8, 29.5)	29.3 (27.8, 30.9)	53,594	3,375	53.6 (53.2, 54.1)	53.1 (51.4, 54.8)
Yes	3,091	430	40.7 (38.2, 43.1)	37.1 (29.2, 45.8)	3,049	422	65.3 (62.8, 67.6)	61.2 (52.4, 69.3)
**Alcohol user**								
No	56,842	3,776	29.4 (29.0, 29.8)	29.4 (27.9, 30.9)	55,907	3,716	53.9 (53.5, 54.3)	53.2 (51.5, 54.9)
Yes	749	83	43.6 (37.8, 49.6)	51.6 (33.9, 68.8)	736	81	64.5 (58.6, 70.1)	76.2 (57.5, 88.4)
**Hypertensive**								
No	47,880	3,138	29.6 (29.2, 30.0)	29.2 (27.6, 30.9)	47,138	3,091	54.0 (53.6, 54.5)	52.4 (50.6, 54.3)
Yes	6,093	405	28.0 (26.9, 29.1)	31.0 (26.1, 36.3)	6,000	400	51.6 (50.3, 52.8)	58.9 (53.3, 64.2)
Missing	3,618	316	30.8 (29.2, 32.4)	31.8 (26.7, 37.4)	3,505	306	57.4 (55.7, 59.1)	57.1 (51.3, 62.7)
**Hyperglycemic**								
No	56,008	3,728	29.6 (29.3, 30.0)	29.6 (28.1, 31.2)	55,100	3,669	54.2 (53.8, 54.6)	53.6 (51.9, 55.3)
Yes	1,000	79	24.1 (21.7, 26.8)	26.0 (17.8, 36.3)	986	78	46.2 (43.2, 49.2)	39.9 (30.1, 50.7)
Missing	583	52	24.9 (21.8, 28.2)	33.4 (21.5, 47.9)	557	50	48.2 (44.4, 52.0)	63.6 (49.1, 75.9)
**Had ≥4 ANC visits**								
No	23,353	1,563	32.6 (32.0, 33.2)	37.2 (34.6, 39.8)	22,991	1,539	56.6 (56.0, 57.3)	59.5 (56.9, 62.2)
Yes	31,718	2,234	27.2 (26.7, 27.7)	24.5 (22.8, 26.4)	31,153	2,196	51.7 (51.2, 52.3)	49.5 (47.4, 51.6)
Missing	2,520	62	29.8 (28.1, 31.6)	45.5 (31.2, 60.6)	2,499	62	57.9 (56.0, 59.8)	56.2 (41.0, 70.2)
**Consumed ≥ 100 IFA**								
No	31,295	2,018	31.8 (31.3, 32.4)	33.3 (31.0, 35.6)	30,786	1,996	56.3 (55.8, 56.9)	58.6 (56.2, 61.0)
Yes	23,776	1,779	26.4 (25.9, 27.0)	26.1 (24.1, 28.1)	23,358	1,739	50.5 (49.9, 51.2)	48.8 (46.5, 51.1)
Missing	2,520	62	29.8 (28.1, 31.6)	45.5 (31.2, 60.6)	2,499	62	57.9 (56.0, 59.8)	56.2 (41.0, 70.2)
**Caesarean section delivery**								

No	45,341	2,993	31.6 (31.2, 32.1)	33.3 (31.5, 35.2)	44,617	2,947	56.3 (55.8, 56.7)	57.7 (55.7, 59.6)
Yes	12,250	866	22.4 (21.7, 23.1)	20.1 (17.7, 22.7)	12,026	850	46.4 (45.6, 47.3)	42.5 (39.4, 45.6)
**Total**	57,591	3,859	29.5 (29.1, 29.8)	29.6 (28.1, 31.1)	56,643	3,797	54.0 (53.6, 54.4)	53.4 (51.7, 55.1)

All n are unweighted.

ANC: antenatal care, ARI: acute respiratory infection, BMI: body mass index, CI: confidence interval, IFA: iron-and-folic-acid, n: sample, OBCs: other backward classes, SCs: scheduled castes, STs: scheduled tribes

FC or children who consumed fish; NFC or children who did not consume fish.

Table S5. Prevalence (%) of anemia among NFC and FC groups of children (aged 6-23 months) by select background characteristics.

	Anemia			
	NFC	FC	NFC	FC
	n	n	% (95% CI)	% (95% CI)
**Age of child (months)**				
6-11	18,560	580	66.4 (65.7, 67.1)	64.4 (60.2, 68.4)
12-23	36,568	3,142	69.3 (68.8, 69.8)	64.7 (62.9, 66.4)
**Sex of child**				
Male	28,506	1,897	69.5 (68.9, 70.0)	64.7 (62.4, 66.9)
Female	26,622	1,825	67.1 (66.6, 67.7)	64.6 (62.2, 66.9)
**Birth order**				
1	21,680	1,256	67.0 (66.4, 67.6)	64.6 (62.0, 67.2)
2	18,336	1,300	67.7 (67.0, 68.3)	64.6 (61.9, 67.2)
3	8,442	547	70.5 (69.5, 71.5)	63.9 (59.4, 68.2)
≥4	6,670	619	72.1 (70.9, 73.2)	65.6 (60.7, 70.3)
**Consumed animal product (excluding fish)**				
No	39,723	419	68.7 (68.3, 69.2)	63.8 (59.1, 68.2)
Yes	15,405	3,303	67.3 (66.6, 68.1)	64.8 (63.0, 66.5)
**Consumed carbohydrate rich food**				
No	21,535	298	67.8 (67.2, 68.5)	63.4 (57.6, 68.9)
Yes	33,593	3,424	68.6 (68.1, 69.1)	64.7 (63.0, 66.4)
**Consumed pumpkin, carrots, Squash (yellow or orange inside)**				
No	43,654	1,223	68.3 (67.8, 68.7)	62.0 (59.2, 64.7)
Yes	11,474	2,499	68.6 (67.7, 69.4)	66.1 (64.1, 68.1)
**Consumed dark leafy vegetables**				
No	38,293	796	68.2 (67.7, 68.7)	59.9 (56.4, 63.2)
Yes	16,835	2,926	68.7 (67.9, 69.4)	66.1 (64.3, 67.9)
**Consumed Fruits**				
No	38,876	654	68.6 (68.1, 69.0)	64.2 (60.3, 67.9)
Yes	16,252	3,068	67.7 (67.0, 68.4)	64.7 (62.9, 66.5)
**Consumed lentils or solid/ semi-solid food**				
No	38,890	616	68.4 (67.9, 68.9)	68.7 (64.9, 72.3)
Yes	16,238	3,106	68.1 (67.4, 68.9)	63.7 (61.9, 65.5)
**ARI**				
No	50,705	3,372	68.1 (67.7, 68.5)	64.4 (62.7, 66.1)
Yes	4,423	350	71.1 (69.8, 72.3)	66.2 (61.3, 70.8)
**Diarrhea**				
No	49,508	3,337	67.9 (67.4, 68.3)	64.4 (62.7, 66.1)
Yes	5,620	385	72.1 (71.0, 73.2)	66.2 (61.4, 70.7)
**Had low birthweight**				
No	41,855	2,881	67.3 (66.8, 67.7)	64.1 (62.2, 65.9)
Yes	9,009	473	71.6 (70.7, 72.5)	65.0 (60.9, 68.8)
Missing	4,264	368	71.8 (70.3, 73.2)	69.7 (63.5, 75.3)
**Household size**				
1-4	14,174	1,093	66.9 (66.1, 67.7)	65.3 (62.2, 68.2)
5-6	20,648	1,492	68.8 (68.2, 69.5)	62.4 (59.8, 64.9)
≥7	20,306	1,137	68.8 (68.2, 69.4)	67.1 (64.2, 70.0)
**Religion**				
Hinduism	41,549	2,042	68.7 (68.2, 69.1)	65.8 (63.8, 67.7)
Islam	7,416	662	67.6 (66.5, 68.6)	66.8 (63.4, 70.0)
Others	6,163	1,018	64.6 (62.7, 66.6)	47.4 (41.3, 53.6)
**Social group**				

SC	11,429	626	69.9 (69.1, 70.7)	71.4 (68.1, 74.5)
ST	10,854	1,288	74.2 (73.0, 75.3)	69.4 (64.6, 73.8)
OBC	21,329	1,100	66.6 (66.0, 67.2)	61.8 (59.2, 64.3)
Others	11,516	708	67.4 (66.6, 68.3)	61.1 (57.8, 64.3)
**Wealth index**				
Poorest	14,555	1,178	73.0 (72.2, 73.7)	74.4 (71.4, 77.1)
Poorer	12,924	865	70.4 (69.6, 71.3)	66.8 (63.2, 70.3)
Middle	11,000	673	67.4 (66.5, 68.3)	63.8 (60.0, 67.4)
Richer	9,348	612	65.1 (64.2, 66.1)	62.7 (58.9, 66.4)
Richest	7,301	394	63.1 (62.1, 64.1)	46.7 (42.3, 51.1)
**Locality of residence**				
Urban	10,697	776	65.0 (64.2, 65.8)	59.2 (56.1, 62.3)
Rural	44,431	2,946	69.5 (69.0, 69.9)	66.7 (64.8, 68.6)
**Regional fish availability**				
Marine water fish	12,140	1,106	65.9 (65.1, 66.6)	58.3 (55.7, 60.9)
Freshwater fish	31,823	2,331	69.1 (68.6, 69.6)	68.9 (66.8, 71.0)
Others	11,165	285	70.5 (69.5, 71.5)	72.3 (64.1, 79.3)
**Age of mother (in years)**				
15-19	2,202	135	71.7 (69.9, 73.4)	85.4 (79.4, 89.9)
20-24	20,453	1,181	69.2 (68.5, 69.8)	70.5 (67.9, 72.9)
25-29	20,373	1,325	67.8 (67.1, 68.4)	61.6 (58.8, 64.3)
30-34	8,551	697	67.1 (66.0, 68.1)	56.3 (52.0, 60.5)
35-49	3,549	384	66.7 (64.9, 68.3)	51.3 (44.7, 57.8)
**Educational attainment**				
No education	10,768	699	73.3 (72.5, 74.2)	74.3 (70.5, 77.7)
Incomplete primary	6,556	452	71.2 (70.0, 72.3)	72.1 (67.1, 76.6)
Incomplete secondary	28,422	1,928	68.2 (67.7, 68.8)	64.9 (62.7, 67.1)
Complete secondary	1,147	75	68.6 (65.9, 71.1)	63.8 (52.8, 73.6)
Higher	8,235	568	60.9 (59.9, 61.9)	51.5 (47.7, 55.4)
**Maternal BMI**				
Underweight	12,357	680	72.1 (71.4, 72.9)	71.4 (68.0, 74.5)
Normal	27,673	1,871	69.2 (68.6, 69.7)	66.1 (63.6, 68.4)
Overweight/obesity	15,098	1,171	63.7 (62.9, 64.4)	58.3 (55.4, 61.2)
**Tobacco user**				
No	52,108	3,304	68.1 (67.7, 68.5)	64.7 (63.0, 66.3)
Yes	3,020	418	76.0 (73.7, 78.1)	64.2 (55.5, 72.1)
**Alcohol user**				
No	54,404	3,643	68.3 (67.9, 68.7)	64.6 (62.9, 66.2)
Yes	724	79	79.6 (74.2, 84.1)	75.0 (56.1, 87.5)
**Hypertensive**				
No	45,976	3,027	68.2 (67.8, 68.6)	64.6 (62.8, 66.3)
Yes	5,815	393	67.7 (66.5, 68.9)	61.8 (56.2, 67.1)
Missing	3,337	302	71.3 (69.7, 72.9)	68.4 (62.7, 73.5)
**Hyperglycemic**				
No	53,949	3,627	68.4 (68.1, 68.8)	64.7 (63.1, 66.3)
Yes	957	70	60.6 (57.6, 63.6)	68.5 (57.3, 77.9)
Missing	222	25	74.5 (68.5, 79.7)	35.3 (17.8, 57.9)
**Had ≥4 ANC visits**				
No	22,352	1,510	69.6 (69.0, 70.2)	67.6 (64.9, 70.1)
Yes	30,353	2,152	67.1 (66.6, 67.6)	62.8 (60.7, 64.9)
Missing	2,423	60	72.2 (70.3, 73.9)	63.8 (48.2, 77.0)
**Consumed ≥ 100 IFA**				
No	29,967	1,944	70.2 (69.7, 70.7)	68.5 (66.1, 70.7)
Yes	22,738	1,718	65.5 (64.9, 66.1)	61.3 (59.1, 63.6)
Missing	2,423	60	72.2 (70.3, 73.9)	63.8 (48.2, 77.0)
**Caesarean section delivery**				
No	43,523	2,894	69.0 (68.5, 69.4)	67.4 (65.5, 69.2)
Yes	11,605	828	66.2 (65.3, 67.0)	57.6 (54.4, 60.7)
**Total**	55,128	3,722	68.3 (67.9, 68.7)	64.6 (63.0, 66.2)

All n are unweighted.

ANC: antenatal care, ARI: acute respiratory infection, BMI: body mass index, CI: confidence interval, IFA: iron-and-folic-acid, n: sample, OBCs: other backward classes, SCs: scheduled castes, STs: scheduled tribes

FC or children who consumed fish; NFC or children who did not consume fish.

Table S6. Association between fish consumption and nutritional status among Indian children (aged 6-23 months).

	Model I ¥ RR / β (95% CI) p	Model II £ RR / β (95% CI) p	Model III € RR / β (95% CI) p	Model IV ∞ RR / β (95% CI) p	Model V µ RR / β (95% CI) p
Severe stunting (RR)	1.049 (0.977, 1.126) 0.189	1.030 (0.953, 1.112) 0.457	0.983 (0.910, 1.061) 0.654	0.987 (0.914, 1.065) 0.728	0.987 (0.914, 1.065) 0.733
Severe wasting (RR)	0.924 (0.824, 1.036) 0.175	1.036 (0.915, 1.174) 0.575	1.013 (0.894, 1.147) 0.845	1.021 (0.902, 1.157) 0.738	1.023 (0.903, 1.159) 0.722
Severe underweight (RR)	0.923 (0.836, 1.020) 0.114	1.040 (0.933, 1.159) 0.478	1.008 (0.905, 1.123) 0.883	1.015 (0.911, 1.131) 0.786	1.017 (0.913, 1.132) 0.761
Hemoglobin level (β)*	2.262 (1.765, 2.759) <0.001	1.776 (1.237, 2.314) <0.001	1.098 (0.567, 1.630) <0.001	1.026 (0.497, 1.555) <0.001	1.030 (0.501, 1.559) <0.001
Mild anemia (RR)	1.001 (0.950, 1.054) 0.970	0.971 (0.918, 1.028) 0.312	0.975 (0.921, 1.032) 0.388	0.976 (0.922, 1.033) 0.398	0.976 (0.922, 1.033) 0.408
Moderate / severe anemia (RR)	0.836 (0.798, 0.877) <0.001	0.879 (0.836, 0.925) <0.001	0.917 (0.872, 0.965) 0.001	0.921 (0.876, 0.968) 0.001	0.920 (0.875, 0.968) 0.001

* OLS (or Ordinary Least Squares) regression analysis was used to estimate the association (β) between fish consumption and hemoglobin levels.

¥ Null model; £ Adjusted for individual characteristics; € Adjusted for individual and household characteristics; ∞ Adjusted for individual, household, and maternal characteristics; and µ Adjusted for individual, household, maternal, and maternal healthcare use characteristics

β: coefficient, RR: relative risk, CI: confidence interval, p: level of significance

Table S7. Association between fish consumption and nutritional status among Indian children (aged 0-59 months).

	Model I ¥ RR / β (95% CI) p	Model II £ RR / β (95% CI) p	Model III € RR / β (95% CI) p	Model IV ∞ RR / β (95% CI) p	Model V µ RR / β (95% CI) p
Stunting (RR)	1.105 (1.069, 1.142) <0.001	1.063 (1.025, 1.103) 0.001	1.036 (0.999, 1.074) 0.058	1.039 (1.002, 1.077) 0.040	1.039 (1.001, 1.077) 0.042
Wasting (RR)	0.889 (0.840, 0.942) <0.001	1.003 (0.942. 1.068) 0.933	1.000 (0.939, 1.065) 0.997	1.005 (0.944, 1.070) 0.876	1.005 (0.944, 1.070) 0.869
Underweight (RR)	0.973 (0.934, 1.013) 0.176	1.030 (0.986, 1.077) 0.181	1.020 (0.977, 1.066) 0.363	1.026 (0.983, 1.071) 0.245	1.026 (0.982, 1.071) 0.247
Any anthropometric failure (RR)	1.007 (0.983, 1.031) 0.580	1.037 (1.011, 1.065) 0.006	1.022 (0.996, 1.048) 0.104	1.025 (0.999, 1.052) 0.059	1.025 (0.999, 1.052) 0.058
Anemia (RR)	0.910 (0.891, 0.930) <0.001	0.931 (0.910, 0.954) <0.001	0.960 (0.938, 0.982) 0.001	0.962 (0.940, 0.984) 0.001	0.962 (0.940, 0.984) 0.001
Severe stunting (RR)	1.062 (1.003, 1.125) 0.041	1.043 (0.979, 1.111) 0.190	1.005 (0.943, 1.070) 0.878	1.007 (0.946, 1.073) 0.820	1.007 (0.945, 1.073) 0.829
Severe wasting (RR)	0.891 (0.811, 0.979) 0.016	1.023 (0.922, 1.134) 0.670	1.016 (0.916, 1.127) 0.762	1.021 (0.920, 1.132) 0.698	1.021 (0.921, 1.133) 0.688
Severe underweight (RR)	0.905 (0.837, 0.979) 0.013	1.020 (0.935, 1.113) 0.651	1.010 (0.926, 1.101) 0.829	1.014 (0.930, 1.105) 0.754	1.014 (0.930, 1.105) 0.749
Hemoglobin level (β)*	1.968 (1.560, 2.375) <0.001	1.521 (1.084, 1.958) <0.001	0.794 (0.364, 1.224) <0.001	0.735 (0.307, 1.163) 0.001	0.741 (0.313, 1.169) 0.001
Mild anemia (RR)	1.059 (1.002, 1.119) 0.042	0.981 (0.925, 1.040) 0.510	0.990 (0.934, 1.050) 0.749	0.990 (0.934, 1.050) 0.747	0.985 (0.930, 1.043) 0.610
Moderate / severe anemia (RR)	0.840 (0.807, 0.874) <0.001	0.885 (0.847, 0.924) <0.001	0.929 (0.890, 0.970) 0.001	0.933 (0.893, 0.974) 0.002	0.932 (0.893, 0.973) 0.001

* OLS (or Ordinary Least Squares) regression analysis was used to estimate the association (β) between fish consumption and hemoglobin levels.

¥ Null model; £ Adjusted for individual characteristics; € Adjusted for individual and household characteristics; ∞ Adjusted for individual, household, and maternal characteristics; and µ Adjusted for individual, household, maternal, and maternal healthcare use characteristics

β: coefficient, RR: relative risk, CI: confidence interval, p: level of significance

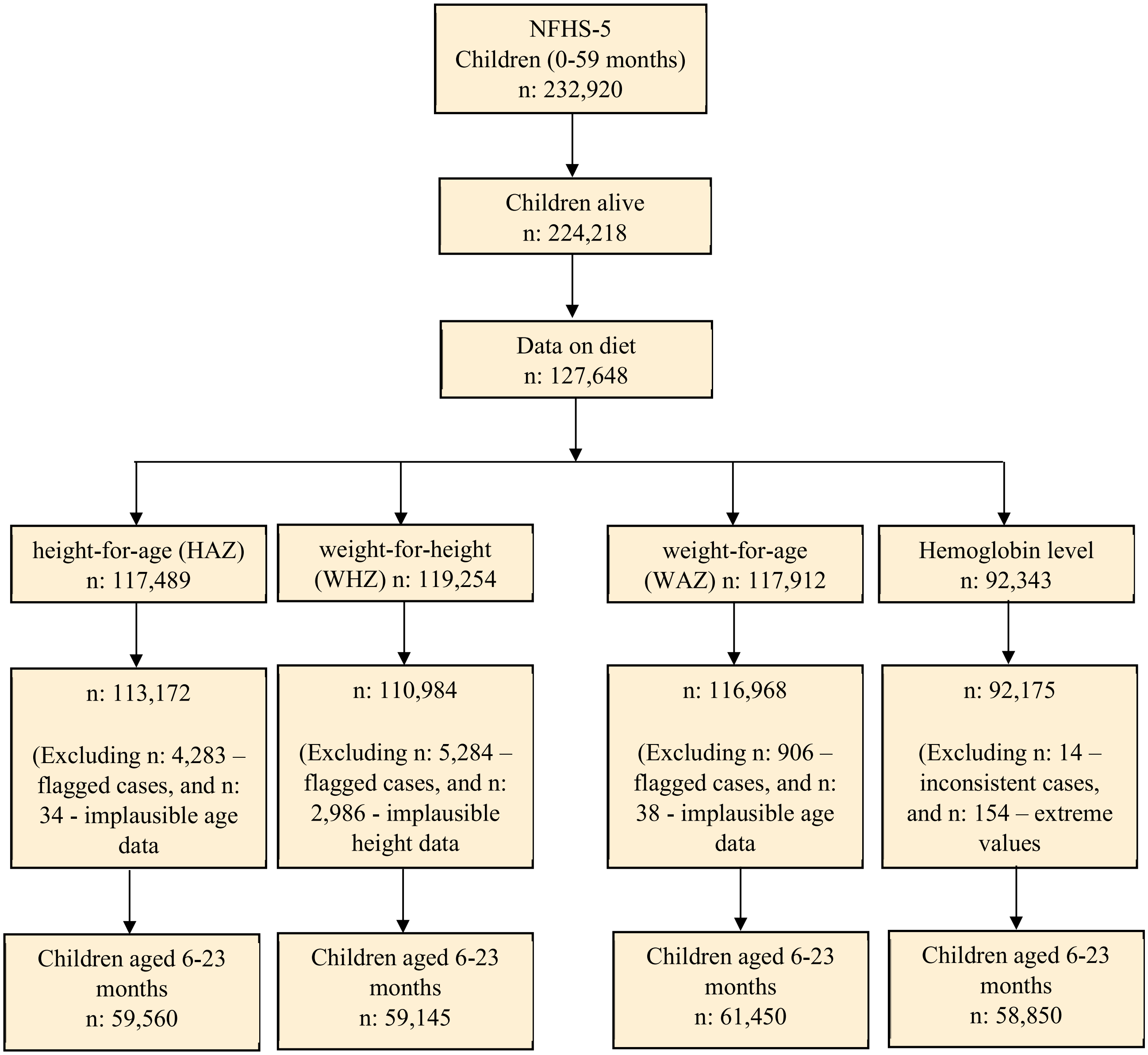

Figure S1. Determining the analytical sample size (n) for child undernutrition indicators: analysis of the 2019-2021 National Family Health Survey (NFHS-5) data.

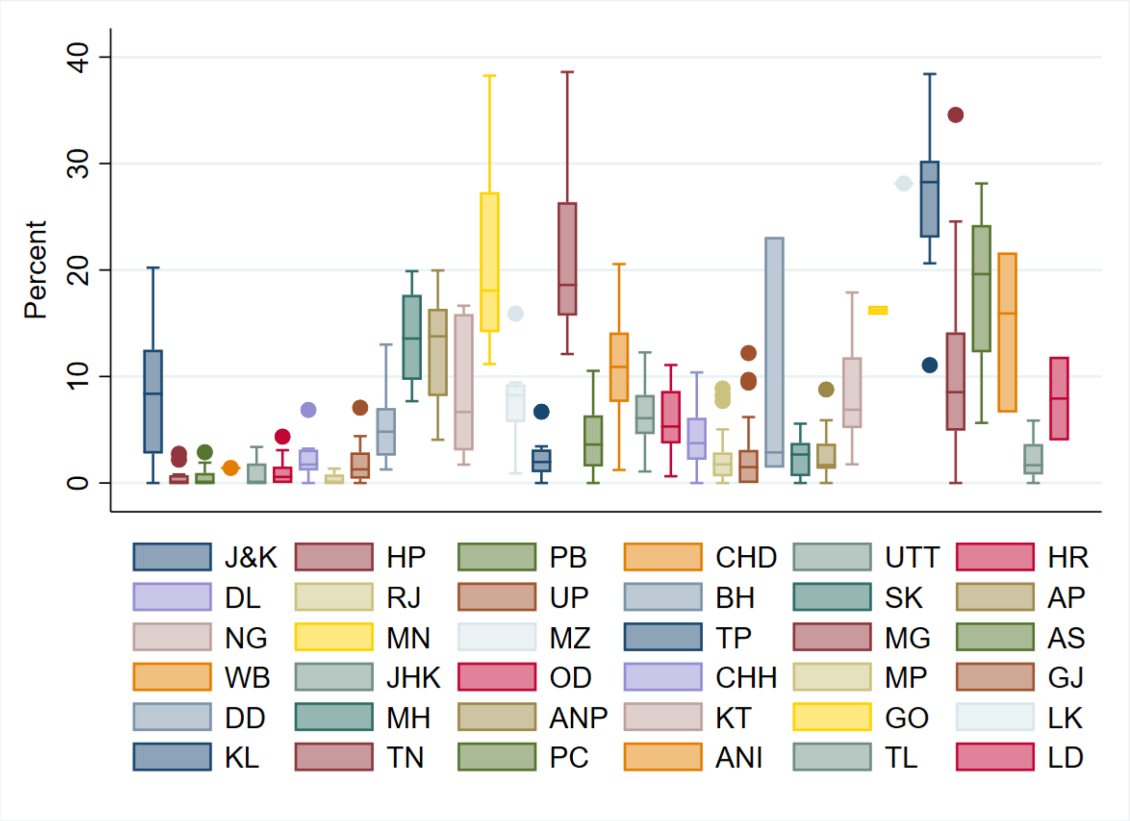

Figure S2. Distribution of the percentage of children who consumed fish in 707 districts across 36 states/Union Territories (UTs), estimated using NFHS-5 (2019–21).

J&K: Jammu & Kashmir, HP: Himachal Pradesh, PB: Punjab, CHD: Chandigarh, UTT: Uttarakhand, HR: Haryana, DL: Delhi, RJ: Rajasthan, UP: Uttar Pradesh, BH: Bihar, SK: Sikkim, AP: Arunachal Pradesh, NG: Nagaland, MN: Manipur, MZ: Mizoram, TP: Tripura, MG: Meghalaya, AS: Assam, WB: West Bengal, JHK: Jharkhand, OD: Odisha, CHH: Chhattisgarh, MP: Madhya Pradesh, GJ: Gujarat, DD: Dadra & Nagar Haveli and Daman & Diu, MH: Maharashtra, ANP: Andhra Pradesh, KT: Karnataka, GO: Goa, LK: Lakshadweep, KL: Kerala, Tamil Nadu, PC: Puducherry, ANI: Andaman & Nicobar Islands, TL: Telangana, LD: Ladakh

The horizontal bar inside the box indicates the median (50th percentile). The lower and upper ends of the boxes represent the 25th and 75th percentiles, respectively, defining the interquartile range (IQR). The bottom 'whisker' below the box is called the lower adjacent value and is equal to the 25th percentile minus 1.5 times the IQR. The upper 'whisker' above the box is called the upper adjacent value and is equal to the 75th percentile plus 1.5 times the IQR. Circles indicate outliers.

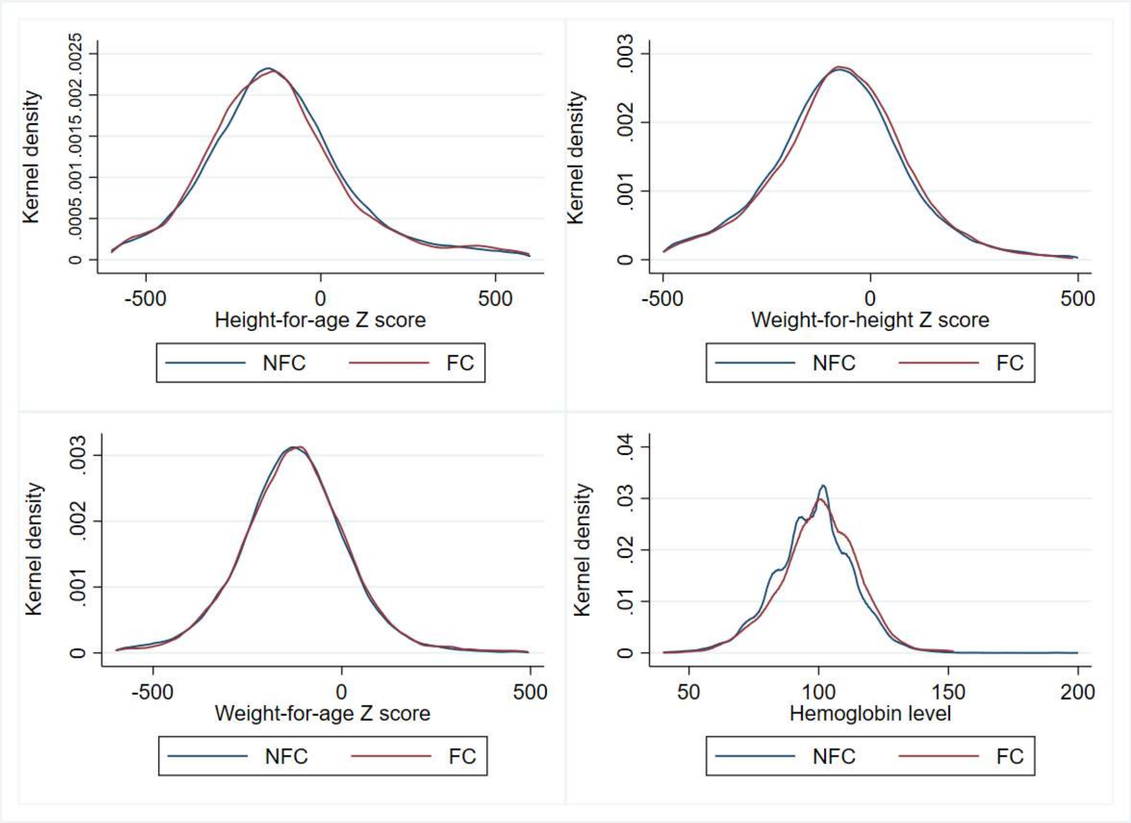

Figure S3. Kernel density distribution of Z-scores for height-for-age (HAZ), weight-for-height (WHZ), and weight-for-age (WAZ), as well as hemoglobin level (g/dl with one decimal place) among children aged 6-23 months, stratified by their fish consumption status – NFC: children who did not consume fish, and FC: children who consumed fish.

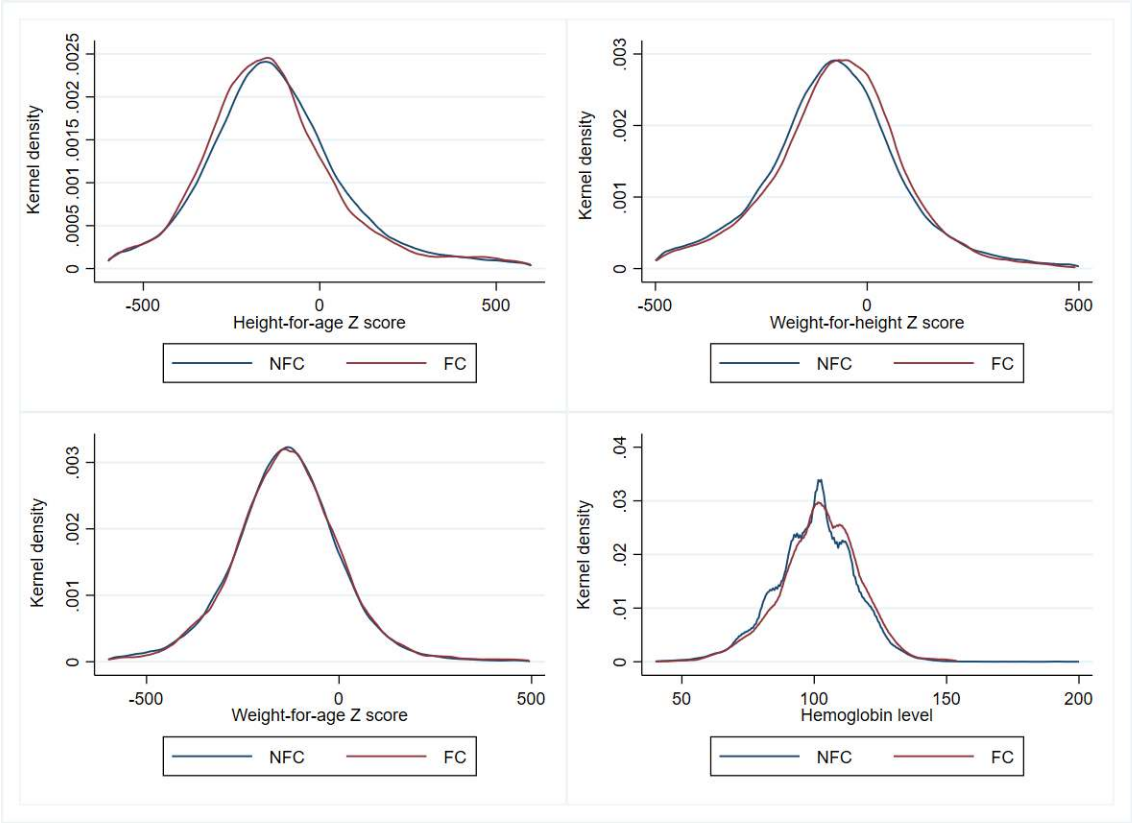

Figure S4. Kernel density distributions of Z-scores for height-for-age (HAZ), weight-for-height (WHZ), and weight-for-age (WAZ) among children aged 0-59 months, as well as hemoglobin level (g/dl with one decimal place) among children aged 6-59 months, stratified by their fish consumption status – NFC: children who did not consume fish, and FC: children who consumed fish.

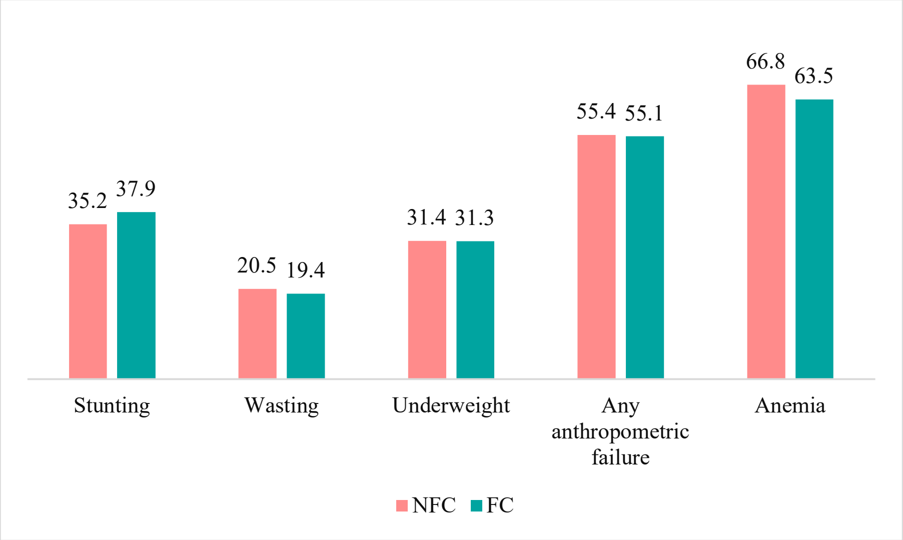

Figure S5. Percentage of children aged 0-59 months exhibiting stunting, wasting, underweight, any anthropometric failure, and anemia, compared by non-fish consumers (NFC) and fish consumers (FC).
